# Utilization of Eye-Tracking Metrics to Evaluate User Experiences—Technology Description and Preliminary Study

**DOI:** 10.3390/s25196101

**Published:** 2025-10-03

**Authors:** Julia Falkowska, Janusz Sobecki, Michał Falkowski

**Affiliations:** 1 Department of Computer Science and Systems Engineering, Wroclaw University of Science and Technology, 50-370 Wroclaw, Poland; janusz.sobecki@pwr.edu.pl; 2 Institute of Control and Computational Engineering, Warsaw University of Technology, ul. Nowowiejska 15/19, 00-665 Warsaw, Poland

**Keywords:** eye tracking, user experience, web design, usability testing, visual attention metrics

## Abstract

This study examines the feasibility of applying eye tracking as a rigorous method for assessing user experience in web design. A controlled experiment was conducted with 102 participants, who interacted with both guideline-compliant websites and systematically degraded variants violating specific principles of Material Design 2. Eleven websites were presented in A/B conditions with modifications targeting three design dimensions: contrast, link clarity, and iconography. Eye-tracking indicators—time to first fixation, fixation duration, fixation count, and time to first click—are examined in conjunction with subjective ratings and expert assessments. Mixed-effects models are employed to ensure robust statistical inference. The results demonstrate that reduced contrast and unclear links consistently impair user performance and increase search effort, whereas the influence of icons is more context-dependent. The study contributes by quantifying the usability costs of guideline deviations and by validating a triangulated evaluation framework that combines objective, subjective, and expert data. From a practical perspective, the findings support the integration of eye tracking into A/B testing and guideline validation, providing design teams with empirical evidence to inform and prioritize improvements in user interfaces.

## 1. Introduction

Creating user-friendly web interfaces requires deep insight into user perception and interaction, not just aesthetic or structural compliance. While standards like ISO 9241-210 [1] emphasize user-centered design through testing and feedback [2,3], traditional UX methods often rely on self-reports or behavior logs, which may fail to capture users’ visual attention and cognitive processes.

Eye tracking offers deeper insight by capturing gaze patterns, fixation points, and saccades, helping identify where users focus, how they navigate content, and where friction occurs [4,5,6]. Despite its value, eye tracking remains underused in comparative design evaluation, particularly for assessing interfaces that only superficially meet modern UI standards.

In this study, we define a well designed interface as one that adheres to Material Design 2 guidelines—featuring high visual contrast, intuitive iconography, clear typography, and well-structured interactive elements. These designs aim to efficiently guide user attention, reduce cognitive load, and support task completion. In contrast, poorly designed variants are intentionally altered to deviate from these principles. They include features such as low contrast, ambiguous or inconsistent icon usage, and vague or misleading call-to-action elements, all of which are expected to disrupt user flow and hinder usability.

We investigate whether eye-tracking metrics can (i) detect and quantify UX differences caused by targeted deviations from design guidelines (reduced contrast, unclear links, missing/ambiguous icons) and (ii) serve as a reliable, practical method for the comparative evaluation of interface variants in A/B testing. Our contributions are threefold. First, we provide an empirical quantification of the usability costs of guideline deviations using eye-tracking metrics. Second, we demonstrate that eye tracking can serve as a practical aid to A/B testing by offering objective and reproducible measures of user effort and performance. Third, and most importantly, we introduce a triangulated evaluation framework that combines eye-tracking data, subjective user ratings, and expert assessments. While prior UX research has typically relied on either eye tracking or questionnaires in isolation, our work explicitly integrates these three perspectives within a controlled A/B design. This approach not only strengthens the validity of findings but also provides a more comprehensive and actionable basis for professional UX evaluation.

## 2. Background

### 2.1. Eye-Movement Recording

Modern methods for recording eye movements include electrooculography, the inductive method, radar-based systems [7,8], and photo-/video-oculography.

Electrooculography (EOG) measures the voltage difference between the retina and cornea via electrodes placed around the eyes [9]. It captures horizontal and vertical movements but is sensitive to lighting changes and skin-related electrical activity, which can introduce noise. Prolonged use may also reduce comfort due to the proximity of electrodes to the eyes.

The inductive method is highly invasive and involves placing a mechanical or optical reference point directly on the eye. Often, special contact lenses with inductive coils are used to detect changes in the electromagnetic field. While accurate, this method is typically unsuitable for long-duration or user-friendly studies.

Radar-based eye tracking uses high-frequency radio waves (e.g., 60–120 GHz) to detect eye movements, eyelid motion, and sometimes gaze direction by analyzing reflected signals. Unlike infrared-based systems, radar can operate in low-light conditions, through glasses, and without a direct line of sight to the eye [7]. However, due to its limited spatial resolution and lack of fixation-level precision, this method is less suitable for UX research requiring detailed eye-movement metrics [8].

Photo- and video-oculography involve recording the motion of the eye using cameras that track specific markers—typically the corneal reflection or pupil. The approach that is most widely used today employs infrared light, which is invisible to users and allows for high precision, non-intrusiveness, and participant comfort. It enables accurate detection of gaze direction by analyzing the light spot reflected off the cornea.

Eye trackers use infrared illumination to generate Purkinje reflections—reflected images from various eye surfaces [10]. Most systems focus on the first and fourth reflections, using their positions relative to the pupil center to estimate gaze direction [11].

Key parameters of eye trackers include the following:

- Sampling frequency (25–2000 Hz), indicating how many gaze data points are recorded per second,
- Accuracy and precision of measurement,
- Head-box size, or the range in which participants can move without losing calibration,
- Monocular vs. binocular tracking,
- Pupil-detection method (bright or dark pupil effect).

The choice of sampling frequency directly influences the temporal resolution of the recorded data. For this study, a frequency of 300 Hz was selected. While lower frequencies (e.g., 60 Hz) can yield similar spatial accuracy for longer fixation events, the higher 300 Hz frequency provides superior temporal resolution. This is critical for precisely capturing the onset and dynamics of rapid eye movements, such as saccades and very short fixations, which are essential for identifying subtle differences in visual search efficiency and cognitive processing during usability tasks [4,11]. For example, a 30 ms saccade and a 200 ms fixation recorded at 60 Hz will yield approximately one sample for the saccade and nineteen for the fixation. At 300 Hz, the same events produce nine and ninety-nine samples, respectively, allowing for a much more precise analysis of oculomotor behavior.

### 2.2. Eye-Tracking in UX

Eye tracking has become a valuable method in studies of human–computer interaction for assessing how users allocate visual attention when performing tasks. It provides objective measures that complement traditional approaches such as surveys, think-aloud protocols, and heuristic evaluations [5,11,12]. In the UX context, eye tracking enables researchers to move beyond subjective impressions and quantify attentional distribution, search efficiency, and cognitive effort.

### 2.3. Visual Attention and Metrics

Visual search relies on alternating fixations and saccades. Fixations indicate where cognitive processing occurs, while saccades represent transitions between focal points. Metrics such as fixation count, fixation duration, time to first fixation (TTFF), and scanpath patterns are widely used to assess user efficiency and attention allocation [13]. These measures allow researchers to assess how interface design facilitates or impedes task completion. A detailed classification based on Holmqvist’s typology is presented in Table 1. In this paper, we focus on TTFF, fixation duration, fixation count, and time to first click as practical indicators of usability.

### 2.4. UX-Evaluation Methods

UX has traditionally been studied using both qualitative and quantitative approaches. Common methods include observation, interviews, usability testing, and standardized questionnaires such as SUS or UEQ. While these provide valuable insights, they are sometimes limited by subjectivity and self-report bias [14]. Eye-tracking offers a complementary approach by generating direct behavioral evidence. We summarize common methods of UX evaluation below:

- Task completion in a working system environment [15,16,17,18],
- Evaluation of specially prepared pages or interface mockups [15,19,20,21,22,23],
- Supplementary use of declarative methods such as surveys and interviews [15,24,25].

### 2.5. Design Guidelines and UX

Design guidelines, such as those codified in Material Design 2, specify principles aimed at ensuring clarity, consistency, and accessibility. Nevertheless, in practice, guidelines may be applied inconsistently or intentionally violated for experimental purposes. Empirical studies show that violations—such as reduced contrast, ambiguous link formatting, or missing icons — can increase user effort and reduce satisfaction [7,9]. Our study, therefore, focuses on these three design aspects, chosen because they represent subtle but impactful variations frequently encountered in real-world web design.

### 2.6. Research Gap

Although prior research has documented general benefits of guideline adherence, fewer studies have directly tested how specific guideline violations manifest in objective eye-tracking metrics. Moreover, while eye tracking is widely used in research, its role as a practical *A/B testing tool* in everyday design workflows has not been fully explored. This paper addresses this gap by systematically manipulating contrast, link clarity, and iconography in otherwise guideline-consistent mock-ups and evaluating the resulting effects on gaze metrics, task performance, and subjective ratings.

## 3. Materials and Methods

### 3.1. Research Goals and Stages

A well designed version of a website should provide users with a superior experience, enabling faster and more efficient task completion. It is expected that metrics related to element visibility, ease of target identification, cognitive effort, and user interest will have more favorable values for the well designed version compared to a poorly designed counterpart. The primary aim of this study is to verify whether eye-tracking metrics recorded during usability tests on websites adhering to design guidelines (version A) are lower than those recorded on websites deviating from these principles (version B). This would validate the practical application of quantitative eye-tracking methods in assessing user experience.

Previous usability studies often focused on isolated website elements without considering their context within the entire site [12]. Jacob and Karn [13] highlighted the need for research involving more realistic user goals:

*“When users search for a tool, menu item, or icon in a typical human-computer interface, they often do not have a clear mental representation of the target. Most literature on visual search assumes participants know the exact target. More basic research is needed on visual search tasks where the target is not fully known. A more realistic search task involves looking for the tool that will help accomplish a specific task, even if the user has not yet seen the tool.”*

Addressing this concern, this study was conducted using several websites, including existing ones and others specifically designed according to current standards. Tasks were designed to reflect the user’s situational context and needs during a site visit (e.g., on an energy provider’s website, receiving an electricity bill and seeking additional information). Importantly, instructions do not direct users to specific page elements or solutions. Versions A (well designed) and B (poorly designed) were created to assess differences in eye-tracking data attributable to guideline deviations, thereby confirming their validity in distinguishing between well designed and poorly designed solutions. The webpages are grouped into three categories: Icons, Links, and Contrast. Each group focuses on design guidelines related to the presentation of icons, links, and contrast parameters, respectively.

The research was conducted in three stages: two pre-tests followed by the main study. Detailed information about each stage is summarized in Table 2. Although the test materials and equipment varied across stages, the overall research methodology remained consistent. This article presents findings from the third stage.

### 3.2. Study-Group Selection

The decision to select a homogeneous group of students for the third stage of the study was made with the aim of minimizing potential confounding variables such as age differences, technical experience, and familiarity with technology that could influence the results. The low variability among participants is confirmed by a low intraclass correlation coefficient (ICC) below 0.2. This approach enables a focused examination of effects specific to the studied technology while reducing the impact of sample heterogeneity on the findings. Specifically, restriction of the sample to computer science students was intended to control for variations in technical skills. While this strategy supports internal validity and methodological control, it also introduces limitations with respect to external validity. The findings should therefore be interpreted with caution when considering more diverse populations.

To enhance the generalizability of the study—for example, by increasing the diversity of technical skills represented—a representative sample of internet users would be necessary. Obtaining such a sample would require identifying key demographic and behavioral characteristics that define the target population. Relevant factors might include age (e.g., 18–65 years), gender, education level, place of residence, frequency of internet use, and experience with digital technologies. Such a sampling strategy would better reflect the demographic composition of internet users, as would be essential for broader generalization of study outcomes.

The preparation of a diverse and representative study group involves the following steps:

- Define demographic and behavioral characteristics that accurately represent the internet-user population, including age, gender, education level, place of residence, frequency of internet use, device types (e.g., smartphones, desktops), and level of internet proficiency.
- Employ stratified sampling techniques to ensure proportional representation of these characteristics within the study group. For instance, if 30% of internet users are aged 18–25, the sample should include a similar proportion. Likewise, if women constitute 50% of internet users, this proportion should be mirrored in the sample.

In summary, conducting research on a homogeneous group enhances internal consistency but inevitably limits the ability to generalize findings. Future studies should therefore expand to include additional groups while maintaining homogeneity in technical skills within each group. For broader generalization, studies should be conducted on samples that accurately represent the internet-user population, with particular attention to varying levels of internet proficiency.

### 3.3. Sample Characteristics

The main study analyzes twelve eye-tracking metrics collected from N=102 participants, examining two types of response variables: time-based and count-based measures. Each of the eleven web pages was presented in two variants (A and B) and classified into one of three project groups: contrast difference, link appearance, and icon presence/absence.

**Figure 1:**
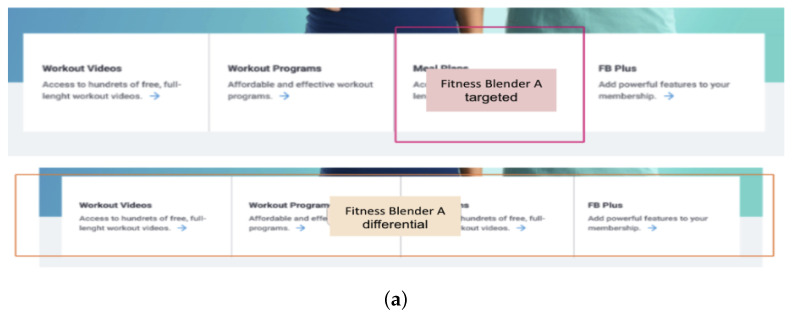

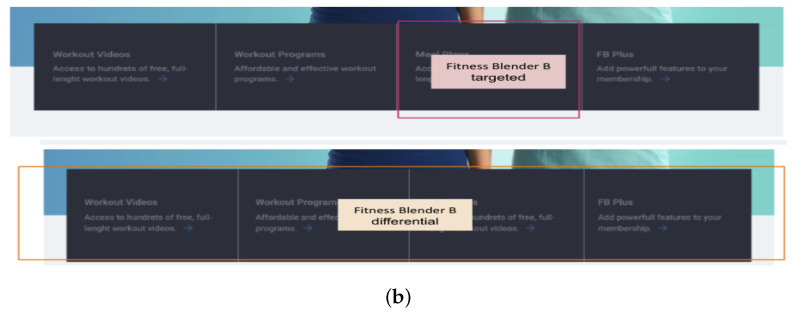
The analyzed Fitness Blender webpage fragments with marked Areas of Interest (AOIs). (**a**) Fitness Blender A; (**b**) Fitness Blender B.

**Figure 2:**
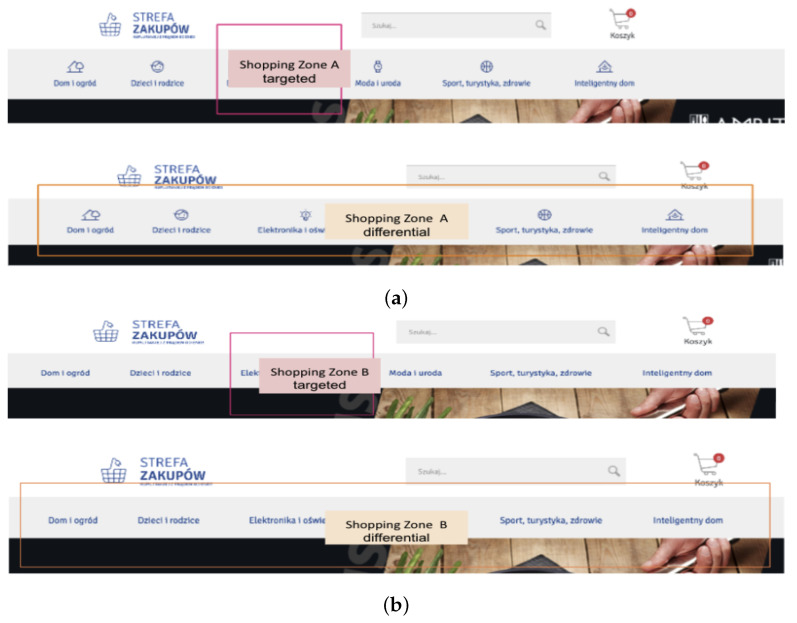
The analyzed Shopping Zone webpage fragments with marked Areas of Interest (AOIs). (**a**) Shopping Zone A; (**b**) Shopping Zone B.

To illustrate the analytical framework, representative examples of Areas of Interest (AOIs) are presented in Figure 1 and Figure 2. In both cases, three AOIs were defined to capture user interaction (see Table 3). The pink region indicates the Target AOI, corresponding to the correct task-solution element. The larger orange region marks the Differential AOI, representing the broader component in which design deviations are introduced. The Search AOI encompasses the remaining page area, excluding the Differential AOI. This structure ensures a consistent mapping of gaze behavior to specific functional elements across all tested websites. While these figures provide illustrative examples, the complete set of AOIs for all eleven websites is available in Supplementary Material S1.

In Table 3, the column “Project Group” indicates the design principle that is intentionally manipulated: Contrast (high vs. low), Icon (presence vs. absence/ambiguity), or Link (clear vs. unclear). The column “AOI” specifies the Areas of Interest defined for eye-tracking analysis: Targeted, the exact element that is the correct solution to the task; Differential, a broader element modified according to the design guideline (e.g., an entire card containing the target); and Search, the rest of the page area outside the differentiating component, reflecting exploratory behavior.

The participant sample consisted primarily of computer science students. While this ensures familiarity with online environments and consistency across participants, it also limits the generalizability of the results to more diverse user groups. This limitation is addressed in Section 5.

### 3.4. Metrics Classification

The research questions and hypotheses were formulated based on the metrics classification employed in user-experience studies by Bojko [4] (see Table 4), alongside the definitions and calculations provided in Tobii Studio version 3.4.8 Enterprise Edition. Assuming that superior user experiences enable faster and more efficient task completion, metrics related to element noticeability, ease of target identification and recognition, cognitive effort, and user interest are expected to reflect shorter times and exhibit lower values for the well designed version of the site (A) compared to the poorly designed version (B).

### 3.5. Research Questions and Hypotheses

The study investigates whether deliberate deviations from Material Design 2 guidelines (contrast, link clarity, iconography) produce measurable differences in user behavior and performance. To avoid fragmentation, hypotheses are grouped by AOI type and metric type rather than listed individually.

Research Question 1 (RQ1). Do guideline deviations increase visual search effort and delay target detection?

- H1a: Time to first fixation (TTFF) on the Target AOI will be longer for poorly designed variants.
- H1b: Fixation count before first fixation on the Target AOI will be higher for poorly designed variants.
- H1c: Total fixation duration within differential AOIs will be greater in poorly designed variants, reflecting increased cognitive effort or confusion.

Research Question 2 (RQ2). Do guideline deviations affect task efficiency and user performance?

- H2a: Time to first click on the Target AOI will be longer for poorly designed variants.
- H2b: Task completion time will be longer for poorly designed variants.
- H2c: Click errors (wrong AOIs) will occur more frequently in poorly designed variants.

Research Question 3 (RQ3). How do different types of deviations (contrast, link clarity, iconography) vary in their impact?

- H3a: Reduced contrast will produce the strongest negative effects on search and performance.
- H3b: Unclear links will increase fixation counts and time to task completion.
- H3c: Icon removal or alteration will show context-dependent effects that are smaller than those associated with contrast or link deviations.

### 3.6. Statistical Methodology

This subsection outlines the statistical tools employed in the analysis and specifies the significance levels used. The significance threshold for all statistical tests was set at α=0.05. For numerical variables, measures of central tendency are reported as the median (Mdn), along with the first (Q1) and third quartiles (Q3). Nominal variables are described by frequencies and their corresponding percentages relative to the total sample size *n* (%).

Differences between two independent groups for numerical variables were evaluated using the Wilcoxon rank-sum test. For comparisons involving two categorical variables, Fisher’s exact test [27] was applied to assess significance.

To estimate the multifactor effects of webpage/project group, AOI (area of interest), and variant on specific metrics, linear mixed-effects models were fitted. Participant ID was included as a random effect. The primary interaction effects were examined via simple contrast analyses, comparing paired differences in estimated marginal means (EMMs). Two model types were used, with the choice depending on the dependent variable: linear mixed models for continuous variables (time-based metrics) and generalized linear mixed models for count variables.

The effects of the categorical variables—webpage, AOI (target element, differentiating component, search area), and variant (A, B)—on time-based metric values were modeled using a multilevel simple change-score approach [28], as expressed in Equation (1):

(1) $$\begin{array}{cc} \mu_{ij} & =\beta_{0}+\beta_{1}\cdot {\mathrm{group}}_{ij}+\beta_{2}\cdot {\mathrm{AOI}}_{ij}+\beta_{3}\cdot {\mathrm{variant}}_{ij}\\ \; & \;+\beta_{4}\cdot {\mathrm{group}}_{ij}\times {\mathrm{AOI}}_{ij}+\beta_{5}\cdot {\mathrm{group}}_{ij}\times {\mathrm{variant}}_{ij}\\ \; & \;+\beta_{6}\cdot {\mathrm{AOI}}_{ij}\times {\mathrm{variant}}_{ij}+\beta_{7}\cdot {\mathrm{group}}_{ij}\times {\mathrm{AOI}}_{ij}\times {\mathrm{variant}}_{ij}+u_{0i}. \end{array}$$

Model fitting was performed using restricted maximum likelihood (REML) with the nloptwrap optimizer [29]. For count-dependent variables, model fitting was conducted via maximum likelihood (ML) using the BOBYQA optimizer [29]. The choice of distribution was guided by tests assessing dispersion in generalized linear models [30]. When no overdispersion was detected, a Poisson distribution was used, with the model defined as follows (Equations (2)–(4)):

(2) $$y_{ij}∼\mathrm{Poisson}\left(\lambda_{ij}\right),$$

(3) $$\begin{array}{cc} \log(\lambda_{ij}) & =\beta_{0}+\beta_{1}\cdot {\mathrm{page}}_{ij}+\beta_{2}\cdot {\mathrm{AOI}}_{ij}+\beta_{3}\cdot {\mathrm{variant}}_{ij}\\ \; & \;+\beta_{4}\cdot {\mathrm{page}}_{ij}\times {\mathrm{AOI}}_{ij}+\beta_{5}\cdot {\mathrm{page}}_{ij}\times {\mathrm{variant}}_{ij}\\ \; & \;+\beta_{6}\cdot {\mathrm{AOI}}_{ij}\times {\mathrm{variant}}_{ij}+\beta_{7}\cdot {\mathrm{page}}_{ij}\times {\mathrm{AOI}}_{ij}\times {\mathrm{variant}}_{ij}+u_{0i}, \end{array}$$

(4) $$u_{0i}∼N\left(0,\sigma_{0}^{2}\right),$$

where λ is the distribution parameter representing both mean and variance. A logarithmic link function ensured the positivity of λ. Model coefficients were interpreted as in Equation (1). When the project group variable instead of the webpage variable was used, Equation (3) was adjusted accordingly.

In cases of overdispersion, a negative binomial distribution was applied [31,32], modifying the model as follows (Equations (5) and (6)):

(5) $$y_{ij}∼\mathrm{Gamma}\text{-}\mathrm{Poisson}\left(\mu_{ij},\alpha \right),$$

(6) $$\begin{array}{cc} \log(\mu_{ij}) & =\beta_{0}+\beta_{1}\cdot {\mathrm{page}}_{ij}+\beta_{2}\cdot {\mathrm{AOI}}_{ij}+\beta_{3}\cdot {\mathrm{variant}}_{ij}\\ \; & \;+\beta_{4}\cdot {\mathrm{page}}_{ij}\times {\mathrm{AOI}}_{ij}+\beta_{5}\cdot {\mathrm{page}}_{ij}\times {\mathrm{variant}}_{ij}\\ \; & \;+\beta_{6}\cdot {\mathrm{AOI}}_{ij}\times {\mathrm{variant}}_{ij}+\beta_{7}\cdot {\mathrm{page}}_{ij}\times {\mathrm{AOI}}_{ij}\times {\mathrm{variant}}_{ij}+u_{0i}, \end{array}$$

where $\mu_{ij}$ denotes the mean (rate) replacing the Poisson parameter λ and α is the shape parameter. Model fitting for these models was conducted using ML and the BOBYQA optimizer [29]. All analyses were performed in R (version 4.1.1; [33]) on a Windows 10 Pro 64-bit system (build 19044), utilizing the following packages: lme4 (v1.1.27.1; [34]), effectsize (v0.8.2; [35]), emmeans (v1.8.2; [36]), sjPlot (v2.8.11; [37]), report (v0.5.1.3; [38]), psych (v2.1.6; [39]), readxl (v1.3.1; [40]), dplyr (v1.0.10), and blmeco (v1.4; [41]).

### 3.7. Research Material

For each of the predefined usability issues, web pages were developed to differentiate between visualization variants representing interaction-effectiveness problems for variants A and B. The pages were designed following the Material Design 2 guidelines [42]. Specifically, the layouts were constructed as collections of Cards, adhering to the design recommendations for the Card component [43]. Cards are user interface (UI) elements—content containers that present information centered around a single topic or piece of content. Typically, a card includes an image or icon, a title, a description, and a call-to-action (CTA) element such as a button or link. Cards are widely utilized due to their adaptability, allowing seamless adjustment to varying content types and screen sizes [44]. As stated in the guidelines, “Each card is made up of content blocks. All of the blocks as a whole are related to a single subject or destination” [43].

**Figure 3:**
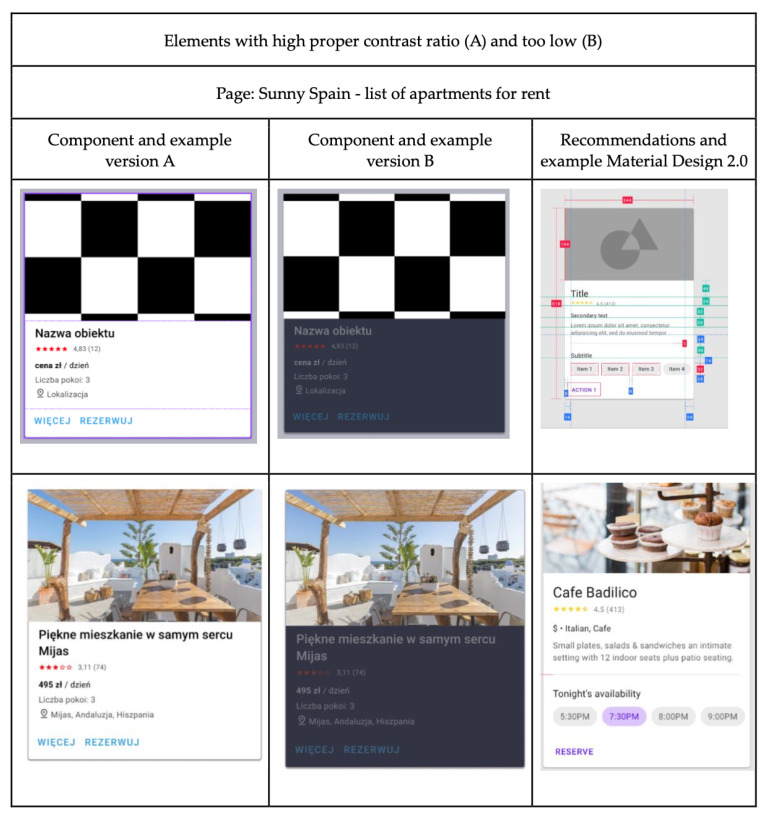
Example of elements with a high, appropriate contrast ratio (A) versus a low contrast ratio (B) on a page displaying a list of apartments for rent.

For illustration, Figure 3 depicts a page designed according to Material Design 2 principles, featuring sets of cards with either an appropriately high contrast ratio (variant A) or a low, insufficient contrast ratio (variant B).

It should be noted that static screenshots were used as stimuli. This choice allowed precise control of content and AOI definitions across participants, thereby increasing internal validity. However, it also reduced ecological validity, since static images cannot fully replicate the interactive and dynamic nature of real web browsing. This limitation is further discussed in Section 5.

### 3.8. Research Course

The study was conducted along two parallel paths, with versions A and B of all 11 website projects alternately presented. In the first path, version A was shown first for website project no. 1 and was followed by version B for project no. 2, and so forth. Conversely, the second path began with version B for website no. 1, then version A for website no. 2, continuing in this alternating manner.

The study followed the procedure outlined below:

1. Welcoming the participant and obtaining signed informed consent. Explaining the study’s procedure and confirming participant understanding.
2. Seating the participant comfortably in front of the eye tracker, ensuring easy access to the mouse and keyboard. Participants were instructed not to ask questions during task performance and to avoid turning their head away from the monitor.
3. Calibrating the eye-tracking device by having the participant remain still and follow a moving dot on the screen with their eyes.
4. Starting data recording using the eye-tracking software.
5. Providing an initial instruction detailing the study procedure (see below).
6. Administering a test task.
7. Conducting the main task while displaying the relevant webpage.
8. Asking a survey question regarding the ease of locating information on the page.
9. Repeating steps 3 and 4 for each webpage included in the study.
10. Ending the recording session in the software.
11. Thanking the participant for their involvement in the study.

Participants provided a single subjective ease-of-use rating after each task. While this measure offers useful complementary insight, it does not cover the full scope of user experience. Standardized UX questionnaires, such as USE or SUS, would have provided a broader and more validated assessment.

#### 3.8.1. Initial Instruction

In this study, you will be required to locate specific information on the presented web pages. A description of the information to be sought will be displayed on the screen, followed by the corresponding webpage. Please search for the requested information on the displayed page and select the element you consider to be the correct solution.

Please note that the pages presented are static images; the elements are not interactive but have been designed to simulate the appearance and layout of a functional website.

The session will commence with a practice task intended to familiarize you with the procedure.

If you have any questions at this stage, please feel free to ask. To proceed to the practice task, press the SPACE key.

#### 3.8.2. Survey Question

Participants were asked: “Is finding information on this page easy?” Responses were recorded using a seven-point Likert scale. The study utilizes a TX300 eye tracker (Tobii Technology AB, Danderyd, Sweden) paired with a 24-inch monitor and a chin rest to stabilize the participant’s head throughout the session. Data collection and processing were conducted using Tobii Studio software, version 3.4.8 Enterprise Edition. The fixation filter employed was the IVT (Identification by Velocity Threshold) filter, which is recommended by the manufacturer. The minimum fixation duration was set to 100 ms. This specific threshold was used because it is a widely accepted and recommended value in eye-tracking research for web-based studies [45]. This setting ensures a robust distinction between meaningful cognitive pauses (fixations) and slower ocular drifts or measurement noise, thereby safeguarding the validity of subsequent metrics that rely on fixation count and duration, such as those quantifying cognitive effort.

### 3.9. Description of Statistical Data Analysis

For the purposes of data analysis, Areas of Interest (AOIs) were defined on elements that were modified according to design guidelines and serve as the target elements for task resolution (designated as the target area). Additionally, many clicks were observed on higher-level modified elements, such as entire cards, rather than on the specific target elements alone. To account for these observations, AOIs were also defined on these broader components (designated as the differentiating component). A third AOI was defined as the remainder of the page area outside the differentiating component that contained the target element.

The selection of appropriate distributions and link functions for the generalized linear mixed models (GLMMs) was determined by a rigorous, multi-step diagnostic procedure to ensure all model assumptions were met and that inferences were robust.

For count-dependent variables, we employed a formal model-selection approach to choose between a Poisson distribution and a negative binomial distribution. This decision was based on testing for overdispersion—a common issue in count data in which the variance exceeds the mean—which would violate a key assumption of the Poisson model. The process was as follows:

1. An initial Poisson GLMM was fitted for each count metric.
2. We then conducted two complementary tests for overdispersion:
  - A likelihood ratio test (LRT) comparing the fit of the Poisson GLMM to a negative binomial GLMM. A significant result (p<0.05) favors the more complex negative binomial model.
  - Calculation of the dispersion statistic (the sum of squared Pearson residuals divided by the residual degrees of freedom). A value significantly greater than 1 indicates substantial overdispersion, necessitating the use of the negative binomial distribution.
3. If either test provided significant evidence of overdispersion (p<0.05 for LRT or dispersion statistic > 1.5), the negative binomial distribution was selected for its ability to model the extra-Poisson variation via a dispersion parameter. Otherwise, the more parsimonious Poisson distribution was retained. The logarithmic link function was used for both distributions to ensure predictions remained within a positive value range.

The overall goodness-of-fit for all final models—both linear mixed models (LMMs) and GLMMs—was assessed by examining the distribution of scaled residuals. For LMMs, we verified the assumptions of normality and homoscedasticity by ensuring residuals were approximately normally distributed (via Q-Q plot inspection) and that their variance was constant across the range of fitted values (via examination of a residual-vs-fitted plot). For GLMMs, we used the simulation-based approach from the DHARMa package to create scaled residuals and perform the same diagnostic checks. In all cases, the residuals exhibited no significant patterns, deviations from normality, or evidence of heteroscedasticity, confirming that the model assumptions were satisfied.

## 4. Results

The results for the three research questions are presented below.

### Hypothesis Verification

The results are reported in line with the three grouped research questions and their sub-hypotheses. Full descriptive statistics, model coefficients, and effect sizes are available in Supplementary Material S2. Table 5 and Table 6 summarize the outcomes of hypothesis testing.

RQ1: Do guideline deviations increase visual search effort and delay target detection?

Consistent with H1a, the time to first fixation (TTFF) on the Target AOI is significantly longer in poorly designed variants across Contrast and Link groups, as shown in Table 5. For H1b, fixation counts before locating the Target AOI are significantly higher in these same groups. H1c is supported in that total fixation duration within Differential AOIs increases under degraded design conditions, indicating that participants expended more effort exploring misleading or unclear elements. Effects for the Icon group are smaller and often not statistically significant, confirming that the impact of iconography is more context-dependent.

RQ2: Do guideline deviations affect task efficiency and user performance?

The results support H2a and H2b, indicating that both time to first click on the Target AOI and overall time to task completion are significantly longer for poorly designed variants (Table 6). Participants frequently require more attempts to identify the correct target, reflecting impaired efficiency. In line with H2c, error rates (e.g., clicks on incorrect AOIs) are also higher in degraded versions, especially when link clarity is reduced. These findings provide strong evidence that guideline deviations increase task difficulty and reduce efficiency.

RQ3: How do different types of deviations (contrast, link clarity, iconography) vary in their impact?

The comparative analyses reveal clear differences between deviation types. Supporting H3a, reduced contrast consistently has the strongest negative impact on TTFF, fixation counts, and time to task completion, indicating significant usability costs. For H3b, unclear links increase both fixation counts and time to task completion, suggesting that link formatting is critical for guiding efficient navigation. H3c is partially supported: modifications of icons produced mixed effects, significant in some tasks but negligible in others. This context-dependent outcome aligns with Section 5, which highlights that users often rely on alternative cues when iconography is ambiguous.

Across the three research questions, the hypotheses were largely supported for Contrast and Link groups, confirming that deviations in these areas significantly hinder user performance and increase search effort. By contrast, the Icon group produced weaker and less consistent effects. These findings demonstrate that eye-tracking metrics reliably distinguish between guideline-compliant and poorly designed web variants, particularly with respect to contrast and link clarity.

## 5. Discussion

### 5.1. Interpreting Findings and Practical Use

This study demonstrates that eye tracking provides a quantifiable, evidence-based method for evaluating the usability costs associated with deviations from established design guidelines. While the negative impact of low contrast or unclear links may seem intuitive, our contribution lies in precisely measuring these effects and validating them through the triangulation of objective gaze data, subjective user ratings, and expert evaluations. The results confirm that these violations impose a significant and measurable cognitive burden that manifests in increased search times, more fixations, and longer decision periods before interaction.

The practical implications of this approach are substantial. Eye-tracking moves beyond subjective preference in A/B testing, providing stakeholders with objective data on how design choices directly impact user efficiency and cognitive load. For instance, consider a web designer deciding between two button designs for a checkout page: one with high contrast (A) and one with lower contrast (B). Our methodology provides a clear, data-driven decision framework:

- If Design B yields a significantly longer time to first fixation on the button, it indicates the element is harder to find, negatively impacting discoverability.
- If users make more fixations on the page before clicking Design B’s button, it suggests higher cognitive effort and a less efficient search path.
- A longer time from first fixation to click for Design B implies user hesitation or uncertainty about the element’s purpose or interactivity.

If Design B performs worse on these metrics, the designer has compelling, quantitative evidence to reject it in favor of Design A, thereby directly improving usability.

For professional web development, these findings offer an empirical basis for prioritizing design revisions. A team can now justify resolving contrast issues before refining iconography, for instance, using evidence that the former has a more dramatic and consistent negative impact on user performance. This facilitates a more strategic allocation of resources and strengthens data-driven decision-making in the design process.

### 5.2. The Divergent Role of Icons

The inconsistent results within the Icon group, where violations sometimes show no effect or even counterintuitive improvements, require specific analysis. This pattern suggests that the role of icons in user-interface design is fundamentally different from that of contrast or link clarity. Unlike the latter, which form the perceptual and functional foundation of usability, icons often act as *supplementary semantic cues*.

Our findings indicate that users engage in a *compensatory strategy* when iconography is ambiguous or missing. They readily pivot to parsing surrounding contextual cues, most notably textual labels, to infer meaning and complete tasks. This adaptive behavior explains why the performance penalties are less severe and more variable for icon modifications. The negative impact of a poor icon is contingent on the clarity and specificity of its accompanying text. On pages where text is highly descriptive (e.g., “Download the report”), the absence of an icon became negligible. Conversely, on pages where text is more abstract, the presence of a clear icon became critical for efficient navigation.

This insight reframes the practical value of icons. They are not indispensable navigational necessities but are powerful secondary elements that enhance scannability, reinforce meaning, and accelerate recognition—but only when their design is intuitive and their meaning is unambiguous. Their value is not absolute but is heavily mediated by the surrounding informational context. This contextual dependency explains why they are less robust predictors in our controlled tests, which intentionally isolate them from other variables.

### 5.3. Order Effects and Task Framing

Page order is counterbalanced to mitigate learning and fatigue effects; participants see both variants with alternating sequences. Task instructions are intentionally broad to avoid biasing attention toward manipulated elements; the analysis focuses on the effect of specific deviations on gaze behavior within this general task frame.

### 5.4. Limitations and External Validity

While this study provides valuable insights, several limitations must be acknowledged to contextualize the findings. First, the use of static mock-ups instead of interactive websites is a necessary methodological choice to ensure precise control over visual variables and AOI definitions across all participants, thereby maximizing internal validity. However, this approach limits the ecological validity of the results, as it does not capture the full dynamic nature of real-world web browsing, including scrolling, hovering, and interactive feedback.

Second, the participant sample consists primarily of computer science students. This homogeneity is intentional; it controls for confounding variables like significant differences in technical proficiency and familiarity with digital interfaces, which allows for a clearer examination of the specific design effects under investigation. The low intraclass correlation coefficient (ICC) confirms the low variability within the sample. However, this choice limits the generalizability of the results to broader, more diverse populations with varying levels of internet literacy, age, or cultural backgrounds. Future studies should aim to replicate these findings with a more representative sample of internet users.

Third, the subjective assessment of user experience is captured by a single ease-of-use rating after each task. While this measure provided useful complementary data, it does not encompass the full spectrum of user-experience constructs (e.g., satisfaction, enjoyment, trust) that are typically measured by standardized, multi-item questionnaires like the SUS or UEQ. Consequently, our understanding of the subjective impact of the design changes is limited to perceived task difficulty.

Finally, the study’s core assumption is that targeted violations of specific design guidelines (contrast, link clarity, iconography) would manifest in objectively measurable oculomotor behavior. The results largely confirm this for contrast and links, but the contextual influence of icons suggests their role is more nuanced and may be dependent on other page elements and users’ compensatory strategies.

### 5.5. Theoretical and Applied Implications

The divergent results for different design elements offer a deeper theoretical insight into user cognition. The strong negative effects of poor contrast and unclear links indicate they create fundamental *perceptual and cognitive barriers*. Low contrast impedes pre-attentive processing, forcing a more effortful and conscious search strategy. Unclear links fail to signal interactivity, violating user expectations and causing hesitation.

In contrast, the ambiguous role of icons suggests they often act as *semantic or reinforcing cues* rather than critical information sources. Users utilize compensatory strategies, relying more heavily on textual labels when icons are missing or confusing. This implies that the necessity of icons is context-dependent and their value is not absolute but is mediated by the surrounding informational context. This suggests that a rigid adherence to usage guidelines for icons may be less critical than ensuring the clarity of fundamental perceptual elements — icons should ideally reinforce meaning and improve scannability, but they are not always a strict requirement for usability when clear textual alternatives are provided.

For future applications, this research provides a validated methodology for using eye tracking as a diagnostic A/B testing tool. It can pinpoint not just *whether* a design is better, but *why* by identifying specific perceptual bottlenecks (e.g., increased search time) or cognitive hesitations (e.g., longer time from fixation to click). Researchers and practitioners can adopt this approach to gain a more granular understanding of user behavior beyond traditional performance metrics.

### 5.6. Future Work

We recommend multimodal evaluation that combines eye tracking with physiological or behavioral signals, systematic studies of additional deviations (e.g., typography, microcopy), and applications to mobile and immersive interfaces. Future studies should address limitation issues by including interactive environments, more diverse samples, and validated questionnaires. Specifically, research should investigate the specific characteristics that make an icon effective (e.g., familiarity, concreteness) and how they interact with text in different contexts to provide more precise design guidance.

## 6. Conclusions

This study makes three primary contributions to the field of user-experience research. First, it provides a validated methodological framework for employing eye tracking in A/B testing, moving beyond simple performance metrics to deliver diagnostic insights into the perceptual and cognitive underpinnings of user behavior. We demonstrate that metrics like time to first fixation, fixation count, and time from fixation to click are sensitive indicators of specific usability issues caused by guideline violations.

Second, our findings offer an empirically grounded hierarchy for design prioritization. The clear, negative impact of contrast and link clarity violations demonstrates that these elements form the foundational layer of usability; their failure creates immediate and significant barriers to user interaction. Conversely, the contextual role of icons suggests they act as a secondary, reinforcing layer. This provides a crucial, evidence-based argument for developers and designers to prioritize resolving perceptual flaws (e.g., contrast ratios) over refining semantic cues (e.g., iconography) when allocating limited resources.

Finally, the study underscores the value of triangulation in UX evaluation. The alignment between objective eye-tracking data, subjective user ratings, and expert assessment creates a compelling, multi-faceted case for design changes that is more persuasive than the evidence of any single method alone.

In practical terms, this work demonstrates that eye tracking can transition from a specialized research tool to a practical asset in the professional design workflow. Its ability to quantify the “why” behind user behavior makes it invaluable for settling design debates, justifying UX debt resolution to stakeholders, and ultimately building more efficient and user-centered digital products. Future work should focus on integrating this approach into interactive and mobile environments and further exploring the nuanced design rules governing elements like icons.

## Figures and Tables

**Table 1:** Detailed classification of metric types with examples based on Holmqvist’s research.

Metric Type	Examples
Eye-movement metrics	Direction (e.g., saccadic direction)
	Amplitude (e.g., scanpath length)
	Duration (e.g., saccade duration)
	Speed (e.g., scanpath speed)
	Acceleration (e.g., saccadic speed skewness)
	Shape (e.g., curvature of giro-saccades)
	Transitions between AOIs (e.g., order of first entries)
	Scanpath comparisons (e.g., sequence correlations)
Gaze-position metrics	Basic position (e.g., fixation coordinates)
	Dispersion (e.g., range, standard deviation)
	Similarity (e.g., Euclidean distance)
	Duration (e.g., fixation duration)
	Pupil diameter
Countable metrics	Saccades: number, proportion, frequency
	Proportion of giro-saccades
	Microsaccade frequency
	Square wave jerk frequency
	Smooth pursuit frequency
	Blink frequency
	Fixation count and rate
	AOI visits: number, proportion, frequency
	Regression and forward/backward gaze frequency
Delay and distance metrics	Onset time (e.g., saccade onset)
	Distance measurements (e.g., between AOIs)

**Table 2:** Summary of conducted studies.

Stage	Number of Participants	Number of Tested Websites	Equipment	Comments
1—Pre-test	22 participants: 9 PhD students and 13 university staff	9	Tobii X2-60 eye tracker (60 Hz) and 15” screen	Conducted at University of Technology Sydney (UTS) from 27.11.2019 to 6.12.2019. Methodology approved by local ethics committee (UTS HREC ETH19-3452)
2—Pre-test	20 participants (students)	7	TX-300 eye tracker (300 Hz) and 23” screen	Conducted at Wrocław University of Science and Technology from 28.06.2021 to 6.07.2021
3—Main study	102 participants (computer science students)	11	TX-300 eye tracker (300 Hz) and 23” screen with chin rest	Conducted at Wrocław University of Science and Technology from 11.11.2022 to 20.12.2022

**Table 3:** List of tested websites divided into project groups and AOIs (Supplementary Material S1).

No.	Website	Project Group	AOI
1	Fitness Blender	Contrast	targeted, differential, search
2	Movies	Icon	targeted, differential, search
3	Green Energy	Icon	targeted, differential, search
4	InPost	Contrast	targeted, differential, search
5	Flower Delivery	Contrast	targeted, differential
6	OLX	Icon	search, targeted, differential
7	Poo-Pourri	Link	search, targeted, differential
8	Salads	Link	search, targeted, differential
9	Sunny Spain	Contrast	search, targeted, differential
10	Movie Database	Icon	targeted, differential, search
11	Recipe Blog	Link	targeted, differential, search

**Table 4:** Metrics classification.

Metric Type	Metric Name in Tobii Studio	Definition (Tobii Studio Manual v3.4.8)	Interpretation
Time	Time to first fixation	Time from stimulus onset to the participant’s first fixation on the area of interest (AOI) (seconds).	Noticeability: indicates how easily and quickly an interface element is noticed. Also reflects the ease of finding the target or the visual accessibility of the element users seek.
Time	Duration of first fixation	Duration of the participant’s first fixation on the AOI (seconds).	Cognitive processing: measures the effort involved in extracting and understanding information.
Time	Fixation duration	Duration of each individual fixation within the AOI (seconds).	Cognitive processing: measures the effort involved in extracting and understanding information.
Time	Total fixation duration	Cumulative duration of all fixations within the AOI (seconds).	Cognitive processing: measures the effort involved in extracting and understanding information.
Time	Visit duration	Duration of each visit within the AOI (seconds). A visit spans from the first fixation on the AOI to the next fixation outside the AOI.	Interest: reflects how long visual attention is maintained after the user initially notices a specific area.
Time	Total visit duration	Total duration of all visits within the AOI (seconds).	Interest: reflects sustained visual attention on a specific area.
Time	Time to first mouse click	Time from stimulus onset to the first left mouse click on the AOI (seconds).	Task completion time.
Time	Time from first fixation to mouse click	Interval between the participant’s first fixation on the AOI and the subsequent mouse click on the same AOI.	Ease of recognizing the target: indicates the effectiveness of communication and clarity of the element’s purpose.
Time	Total saccade time during visits	Calculated as total visit duration minus total fixation duration.	*Proposed metric (not in Bojko’s classification). Based on Kotval and Goldberg [26], a greater number of saccades may indicate a less efficient search for information.
Count	Number of fixations before	Number of fixations before the participant first fixates on the AOI (count).	Noticeability: indicates how quickly and easily an element is noticed, reflecting ease of finding the target.
Count	Number of fixations	Number of fixations within the AOI (count).	Interest: indicates the duration of visual attention on the area after initial notice.
Count	Number of visits	Number of visits to the AOI. Each visit spans from the first fixation on the AOI to the next fixation outside it.	Ease of recognizing the target: reflects how effectively the element communicates its purpose.

**Table 5:** Results of hypothesis testing for RQ1 (visual search effort and target detection) across guideline deviations. The table reports effects for time to first fixation (TTFF), fixation count, and total fixation duration within defined AOIs. Comparisons are made between well designed (Version A) and poorly designed (Version B) variants for each Project Group (Contrast, Link, Icon).

		Contrast					Link		
Eye-Tracking Metrics	AOI	Fitness Blender	InPost	Flower Delivery	Sunny Spain	Contrast Group	Poo-Pourri	Salads	Link Group
Time to first fixation	targeted						A<B		
	differential								
Time to first fixation	targeted								
Fixation time	targeted					A<B		A>B	A>B
Total fixation time	Search		**A<B**			A<B	**A<B**		A<B
Visit time	targeted			A<B					
	Search						**A<B**		
Total visit time	Search		**A<B**			A<B	**A<B**		A<B
Time from first fixation to mouse click	targeted		A<B				A<B		A<B
	differential		A<B			A<B	A<B		A<B
Total saccade time during a visit	differential						A<B		
	Search		**A<B**				**A<B**		A<B
Number of visits	differential		A<B		A<B	A<B			
	Search		**A<B**				**A<B**		
Number of fixations	targeted	A>B					A<B		
	differential						A<B		
	Search		**A<B**		A>B		**A<B**	A>B	A<B
Number of fixations before	targeted	A<B	A<B	A<B	A>B				
	differential				A>B				

Time to complete the task	n/a		A<B				A<B		
Respondents’ score of the ease of task completion	n/a		A>B				A>B		

**Legend:** green: accepted hypothesis, red: significant contrast result inconsistent with the hypothesis. **Note:** “A<B” indicates the metric value for variant A is significantly lower than for variant B; “A>B” indicates it is significantly higher.

**Table 6:** Results of hypothesis testing for RQ2 and RQ3 (task efficiency and variation across deviation types). The table reports effects for time to first click, time to task completion, and click error rates. Comparisons are made between well designed (Version A) and poorly designed (Version B) variants, with outcomes summarized by Project Group (Contrast, Link, Icon).

		Icon					
Eye-Tracking Metrics	AOI	Filmy	Green Energy	OLX	Shopping Zone	Tchibo	Icon
Time to first fixation	targeted		A>B				
	differential		A>B				
Time to first fixation	targeted						A<B
Fixation time	targeted	A>B	A<B	A<B			
Total fixation time	Search						
Visit time	targeted						
	Search		A>B				
Total visit time	Search						
Time from first fixation to mouse click	targeted	A<B					
	differential	A<B					
Total saccade time during a visit	differential	A<B					
	Search	A<B					
Number of visits	differential						
	Search						
Number of fixations	targeted						
	differential	A<B					
	Search				A>B		
Number of fixations before	targeted		A>B	A<B	A<B	A>B	
	differential		A>B	A<B			

Time to complete the task	n/a						
Respondents’ score of the ease of task completion	n/a						

**Legend:** green: accepted hypothesis, red: significant contrast result inconsistent with the hypothesis. **Note:** “A<B” indicates the metric value for variant A is significantly lower than for variant B; “A>B” indicates it is significantly higher.

## Data Availability

Data supporting the findings of this study are available from the corresponding author (J.F.) on reasonable request.

## Supplementary Materials

The following supporting information can be downloaded at: https://www.mdpi.com/article/10.3390/s25196101/s1, Supplementary material S1. The analyzed Areas of Interest (AOI) on the examined websites; Supplementary material S2. Results of the statistical analysis.
