# Supplementary material for: Utilization of Eye-Tracking Metrics to Evaluate User Experiences—Technology Description and Preliminary Study
Source: Sensors (Basel). 2025 Oct 3;25(19):6101. doi: 10.3390/s25196101 (PMC12526729; doi:10.3390/s25196101)
Supplement: Supplementary file 1 [file sensors-25-06101-s001.zip › Supplementary material S1.pdf]

Supplementary material S1. The analyzed Areas of Interest (AOI) on the examined websites.

The analyzed webpage fragments with marked Areas of Interest (AOI): pink indicates the target element area, while the larger orange-marked component represents the differential area.
The search area encompassed the entire page, excluding the differential area.

<table>
<tr><th>Page name</th><th>The analyzed webpage fragments with marked Areas of Interest (AOI)</th></tr>
<tr><td>Fitness Blender A</td><td>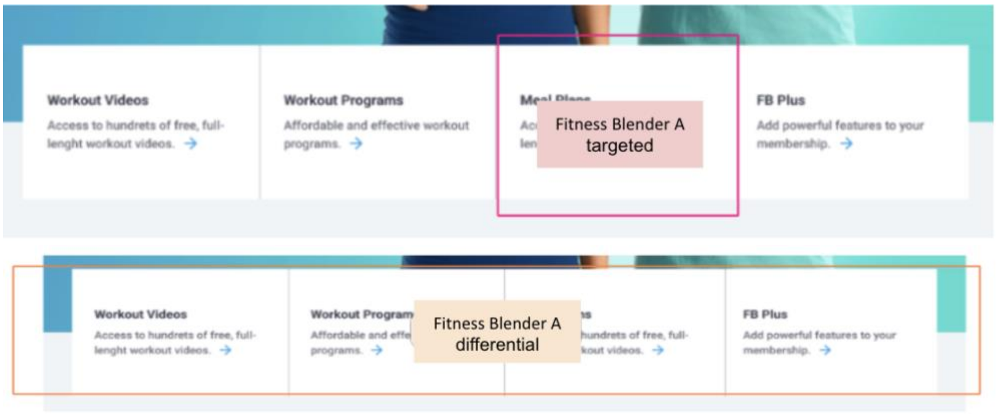
</td></tr>
<tr><td>Fitness Blender B</td><td>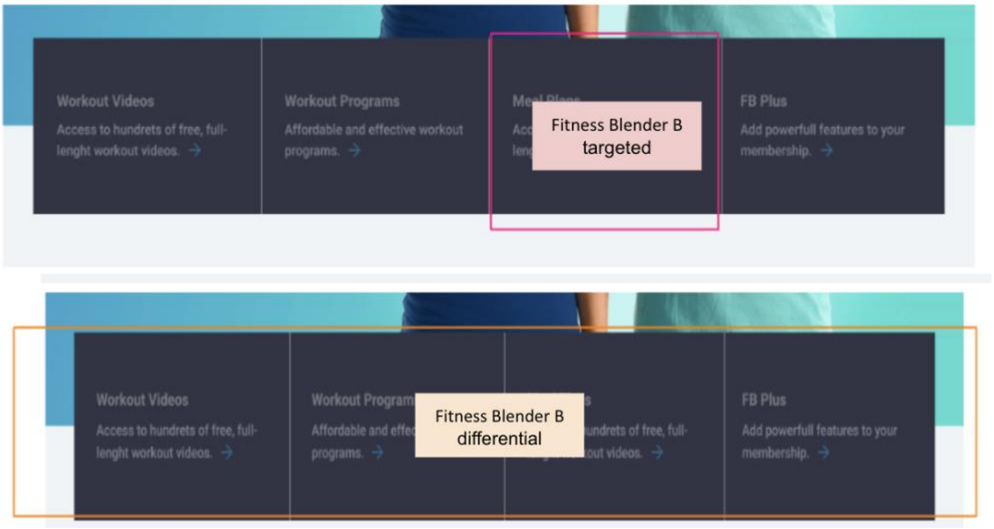
</td></tr>
<tr><td>Films A</td><td>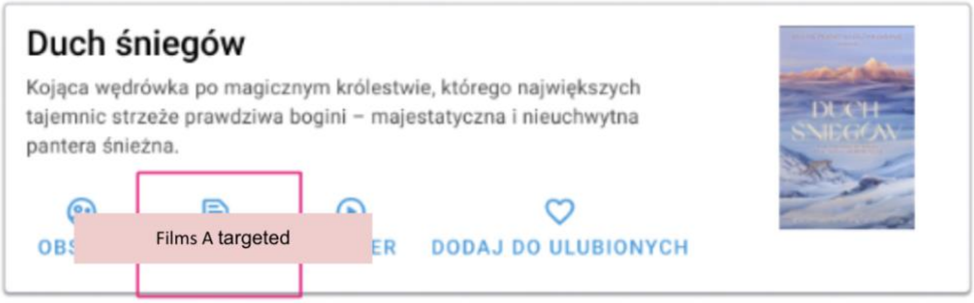
</td></tr>
</table>

<table>
<tr><td></td><td>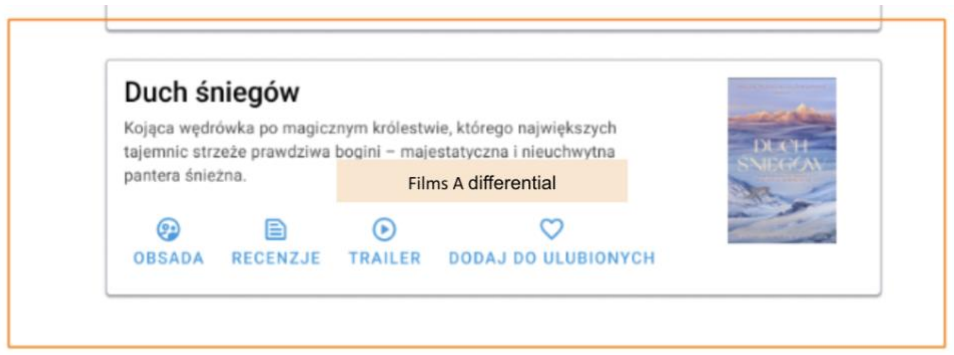
</td></tr>
<tr><td>Films B</td><td>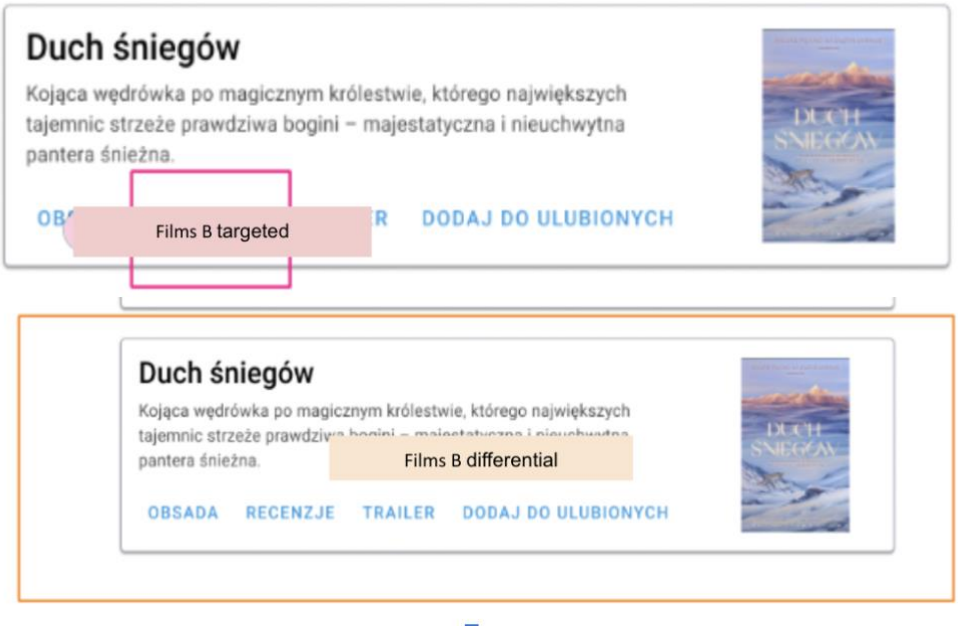
</td></tr>
<tr><td>Green Energy A</td><td>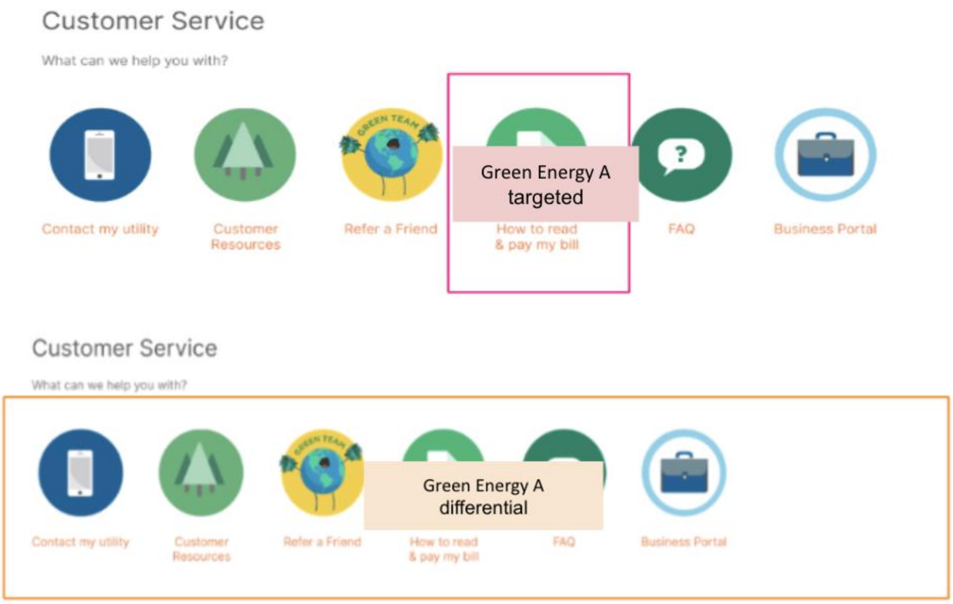
</td></tr>
</table>

Green Energy B	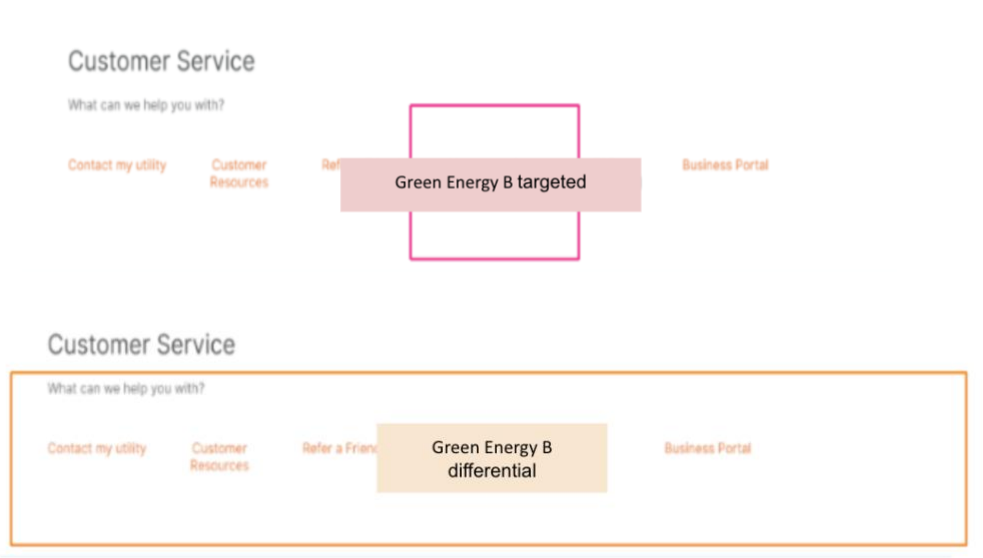
InPost A	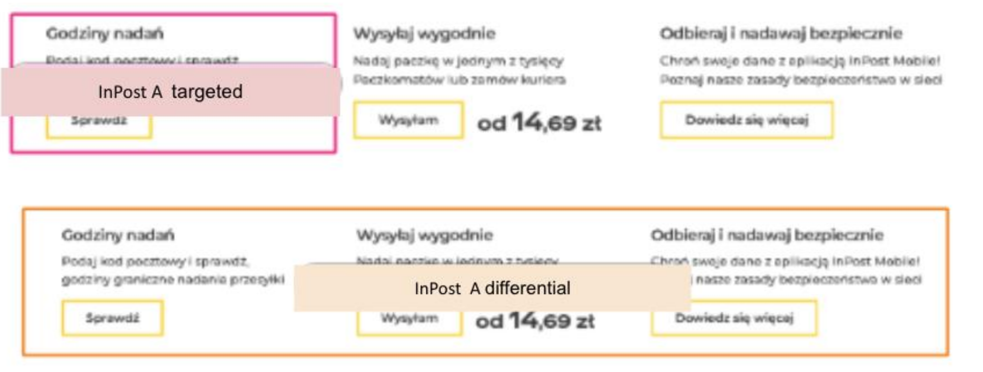
InPost B	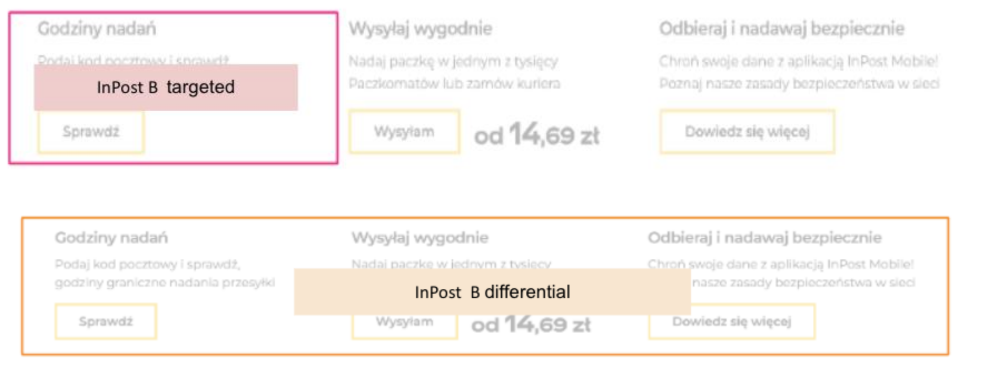

Flower Delivery A	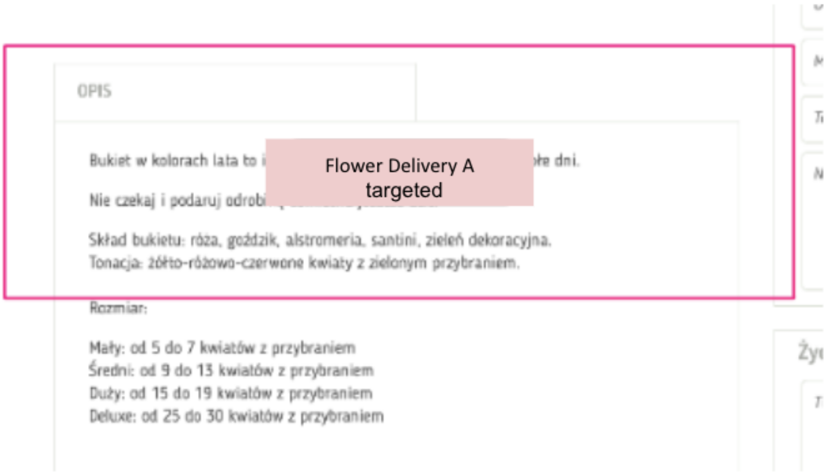
Flower DeliveryB	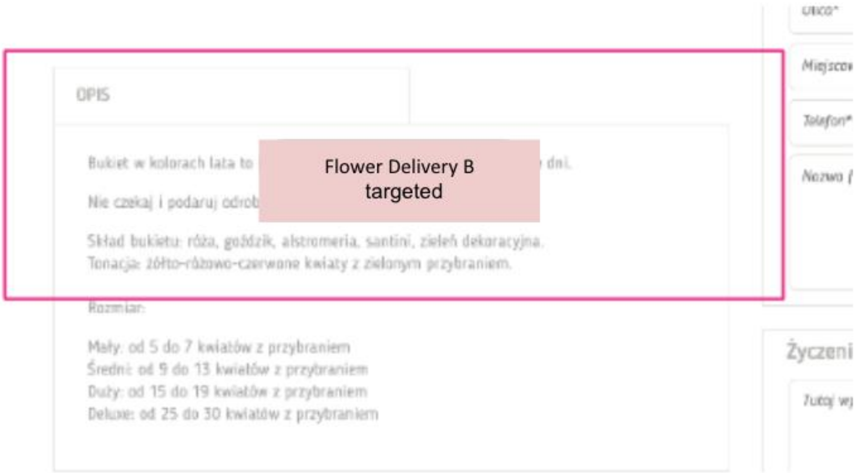
OLX A	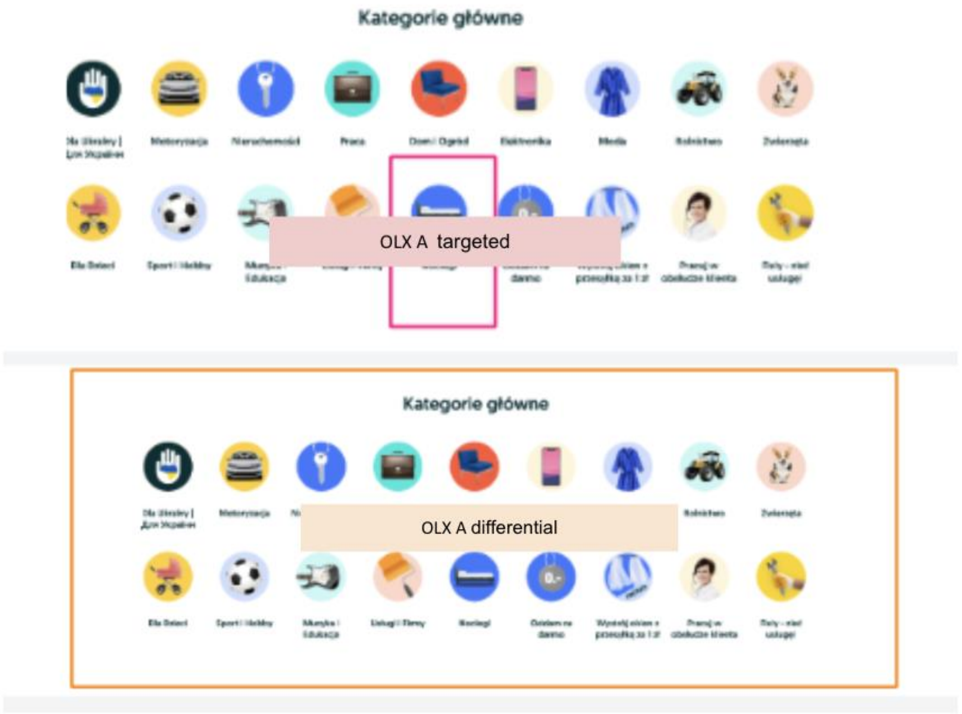

OLX B	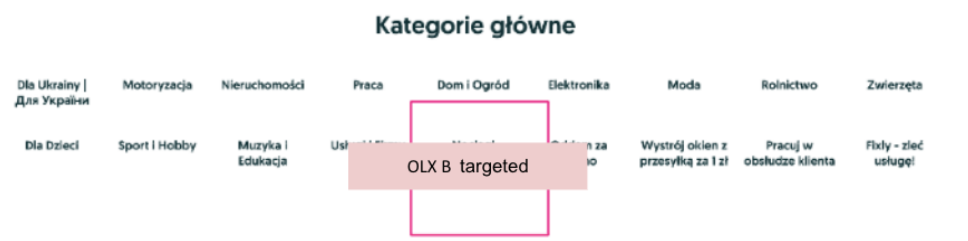<br>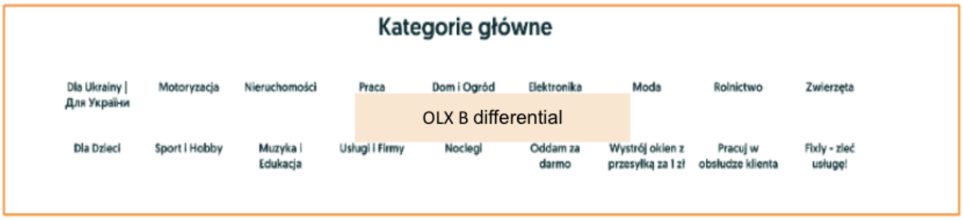
Poo-Pourri A	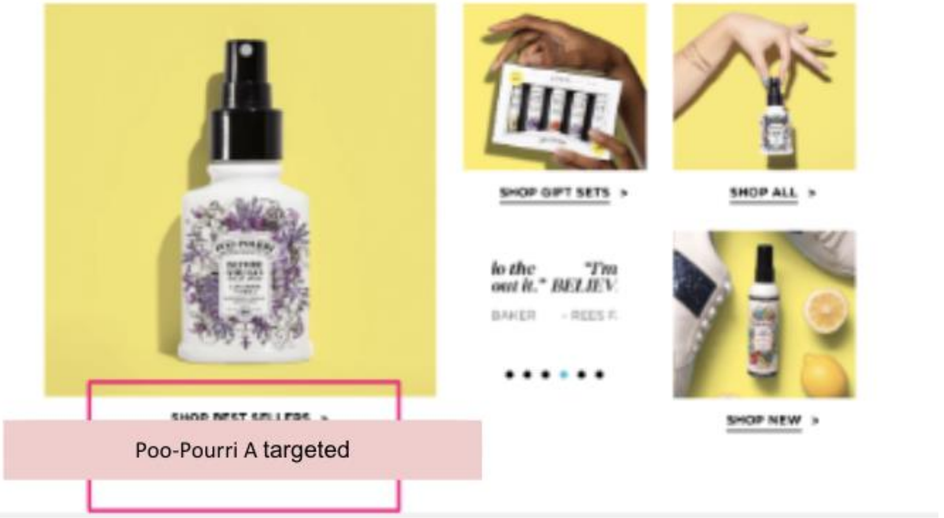<br>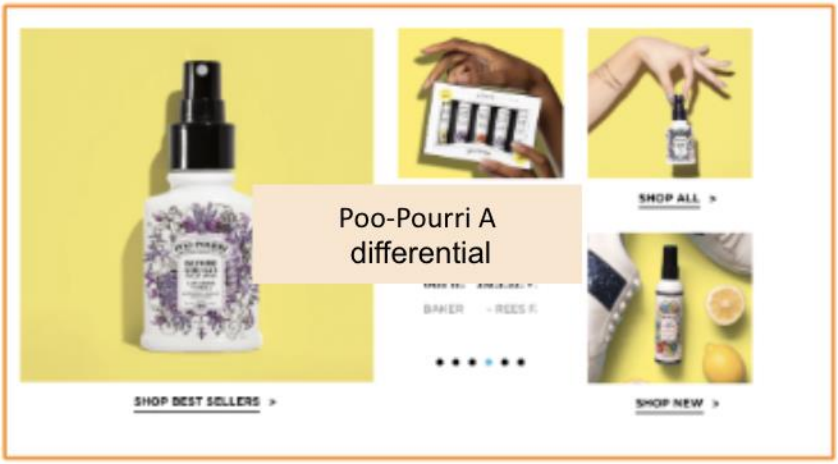

<table>
<tr>
<td>Poo-
Pourri B</td>
<td>
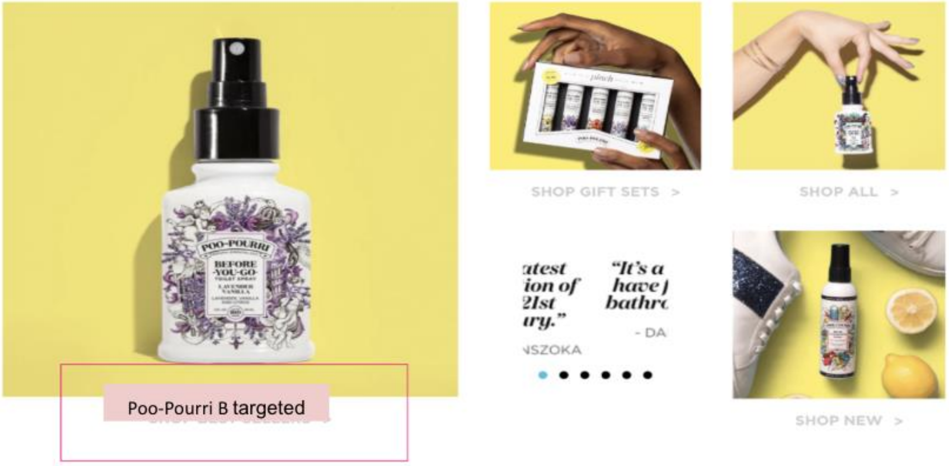

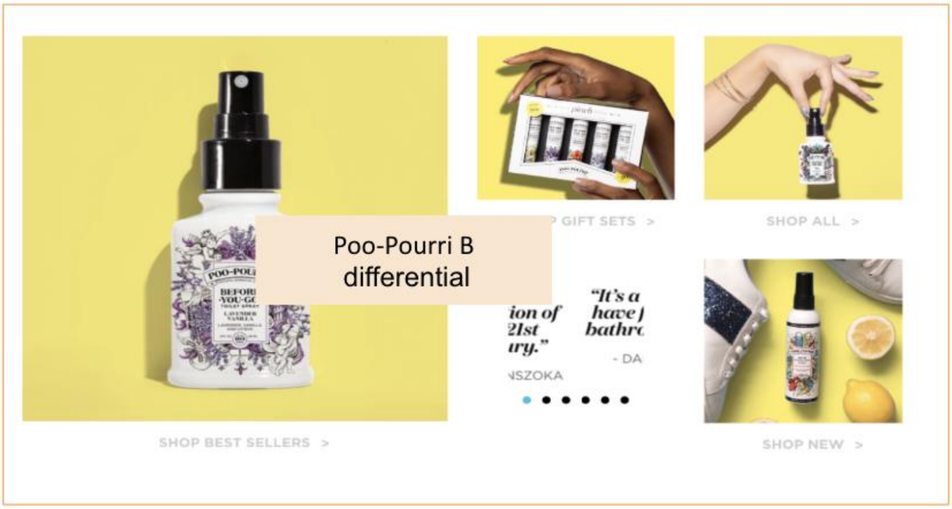

</td>
</tr>
<tr>
<td>Salads A</td>
<td>
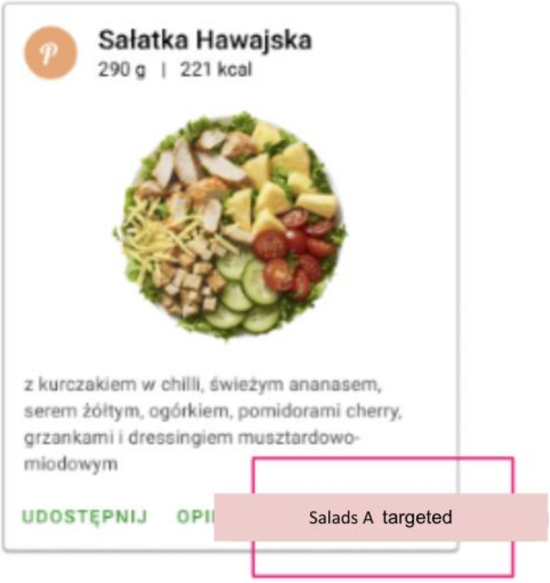

</td>
</tr>
</table>

<table>
<tr>
<td></td>
<td>
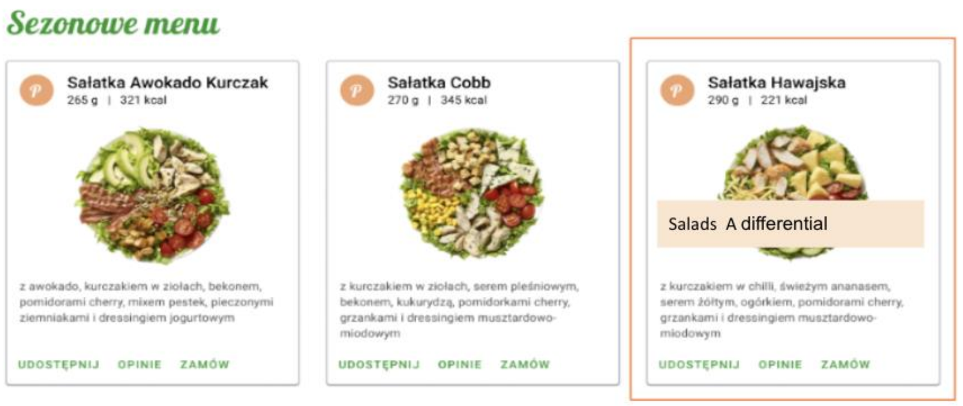

</td>
</tr>
<tr>
<td>Salads B</td>
<td>
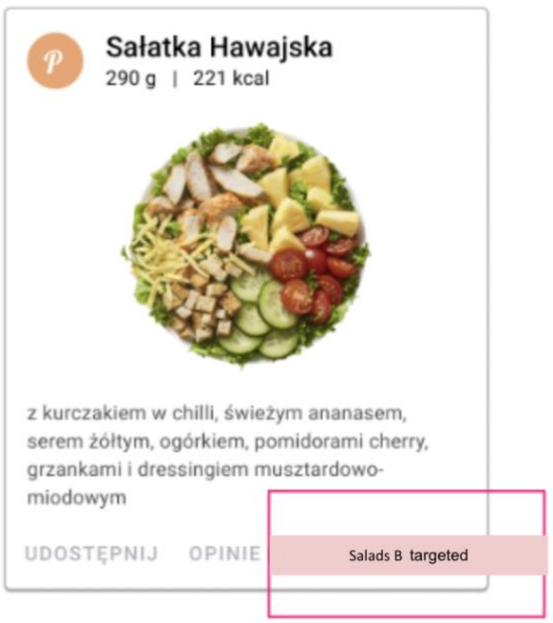

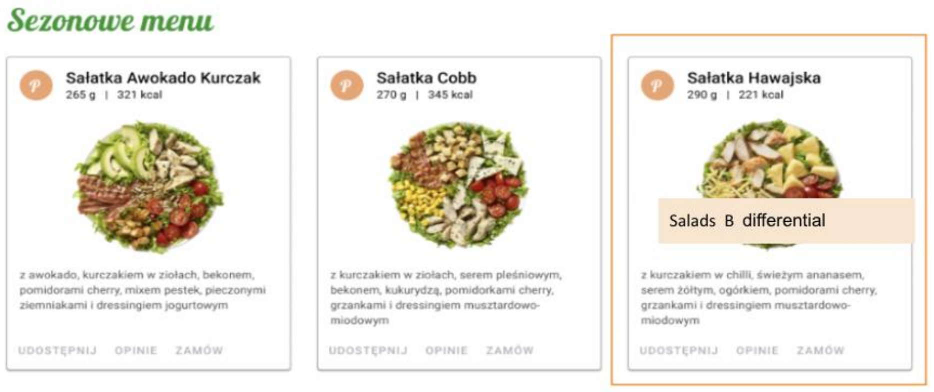

</td>
</tr>
</table>

Sunny Spain A

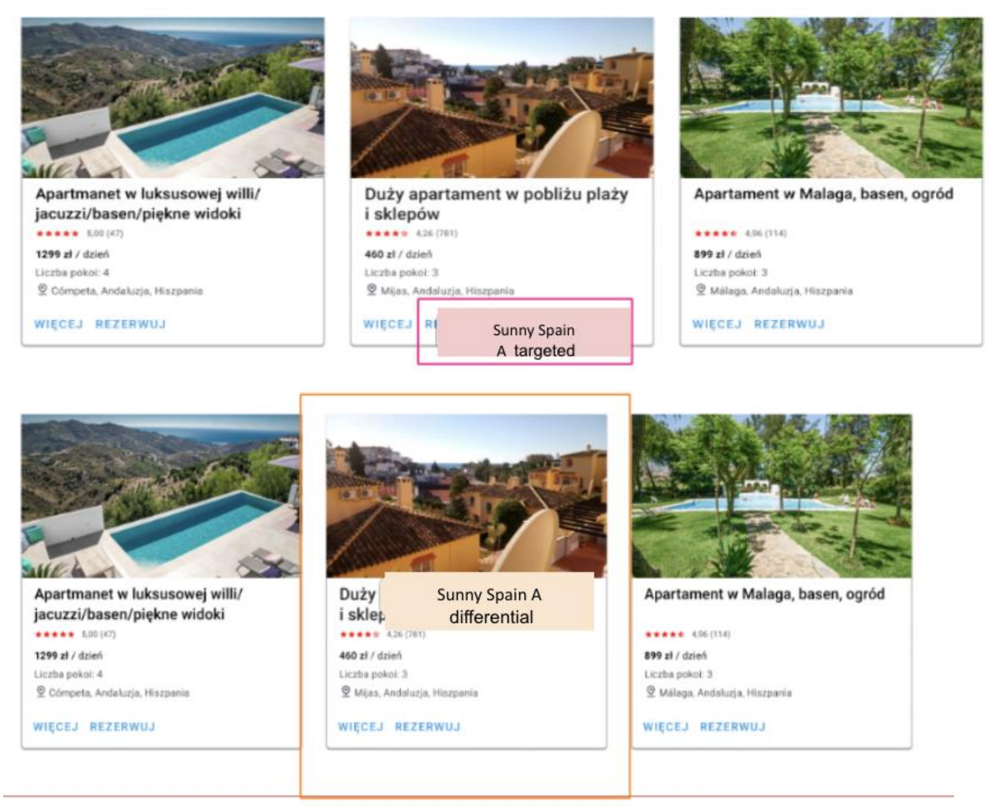

Sunny Spain B

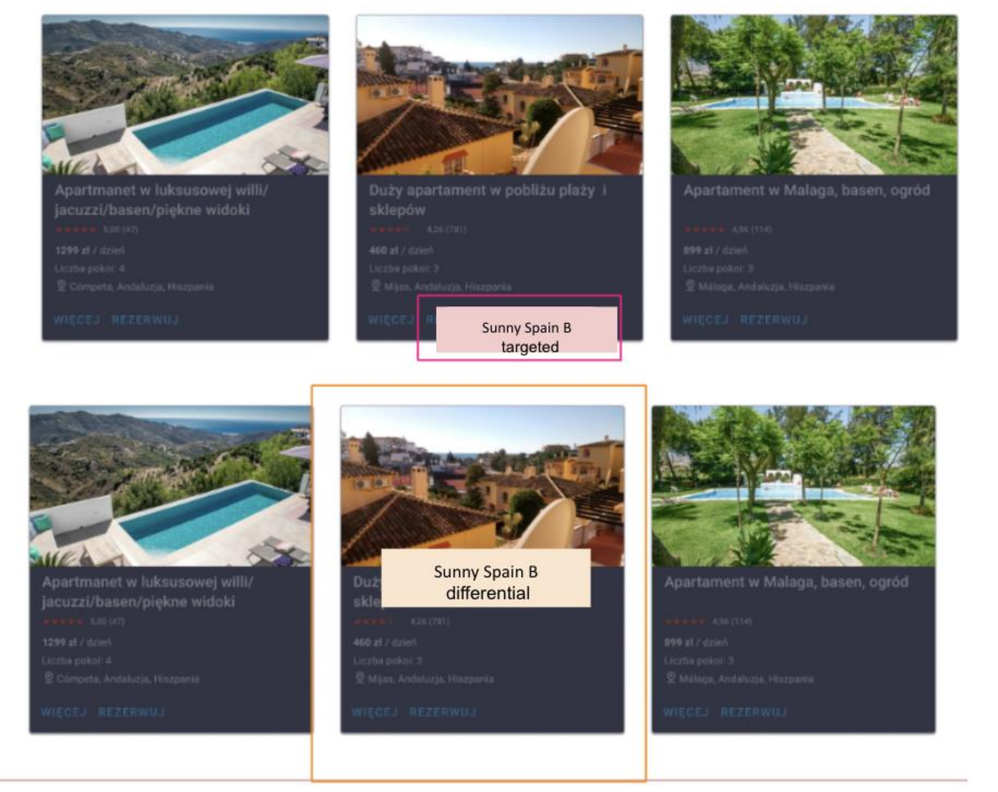

<table>
<tr><td>Shopping Zone A</td><td>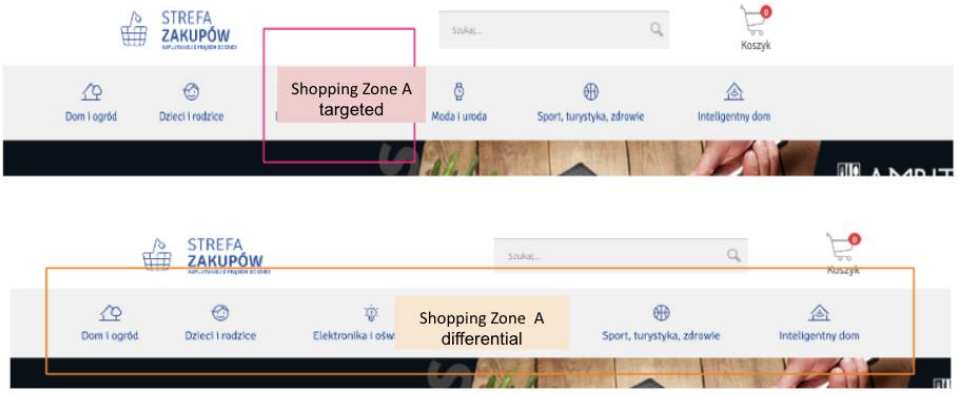
</td></tr>
<tr><td>Shopping Zone B</td><td>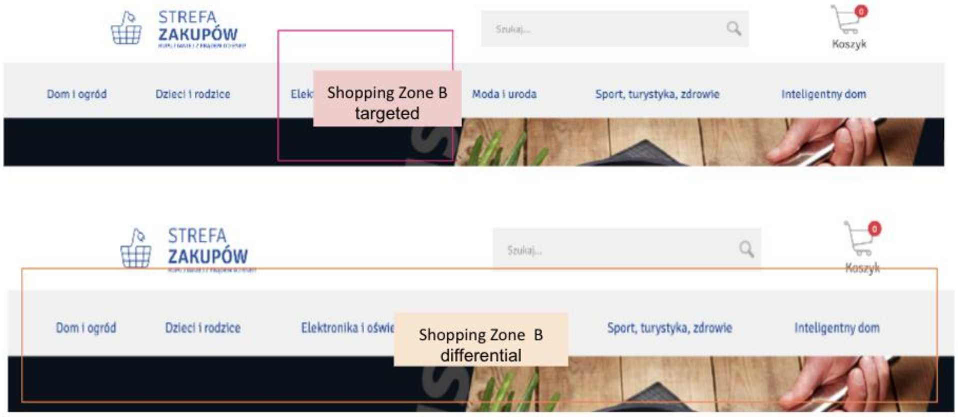
</td></tr>
<tr><td>Tchibo A</td><td>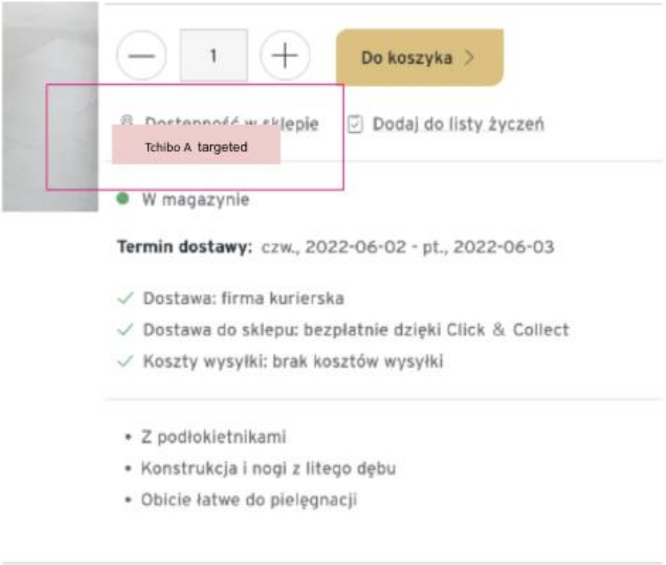
</td></tr>
<tr><td>Tchibo B</td><td></td></tr>
</table>

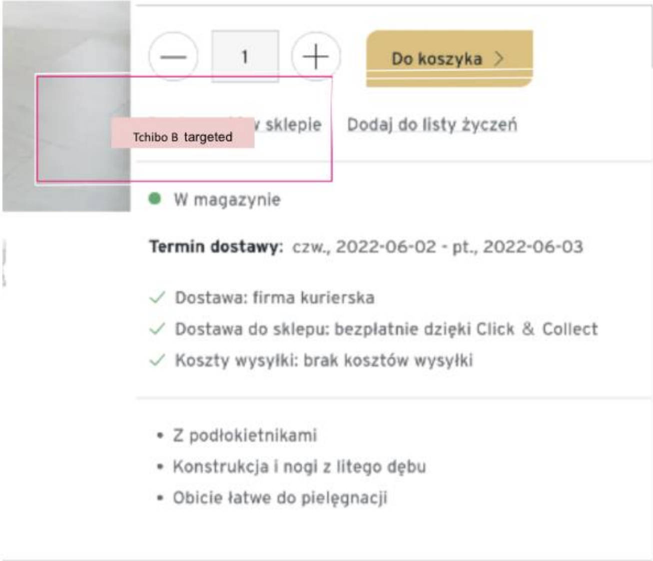
1
Do koszyka
Tchibo B targeted
sklepie
Dodaj do listy życzeń
W magazynie
Termin dostawy: czw., 2022-06-02 - pt., 2022-06-03
Dostawa: firma kurierska
Dostawa do sklepu: bezpłatnie dzięki Click & Collect
Koszty wysyłki: brak kosztów wysyłki
Z podłokietnikami
Konstrukcja i nogi z litego dębu
Obicie łatwe do pielęgnacji
