# Supplementary material for: Utilization of Eye-Tracking Metrics to Evaluate User Experiences—Technology Description and Preliminary Study
Source: Sensors (Basel). 2025 Oct 3;25(19):6101. doi: 10.3390/s25196101 (PMC12526729; doi:10.3390/s25196101)
Supplement: Supplementary file 1 [file sensors-25-06101-s001.zip › Supplementary material S2.pdf]

Supplementary material S2. Results of the statistical analysis.

The results of the contrast analysis for p<0.05 and the corresponding marginal mean (EMM) estimates are shown below. In the tables, bold indicates the area of interest for which the value is significantly larger. If the contrast score is positive, it is the first element, if negative, it is the second element.

1. **1.** Eyetracking metrics

1.1. Time to first fixation

1.1.1. *Pages from the project group Icon*

Table S1. Results for the page from the Icon project group. Time to first fixation (1).

webpage	*contrast*	*result*	*SE*	*df*	*95% CI*	*p*
Green Energy	A targeted - B targeted	2.02	0.81	1200.77	[0.42, 3.61]	0.013
	A differential - B differential	2.40	0.81	1200.77	[0.81, 4.00]	0.003

Table S2. Results for the page from the Icon project group. Time to first fixation (2).

webpage	*variant*	*AOI*	*EMM*	*SE*	*df*	*95% CI*
Green Energy	A	targeted	11.49	0.56	1206.57	[10.39, 12.59]
	B	targeted	9.48	0.59	1195.38	[8.32, 10.63]
	A	differential	8.55	0.56	1206.57	[7.45, 9.65]
	B	differential	6.15	0.59	1195.38	[4.99, 7.30]

1.1.2. *Pages from the Link project group*

Table S3. Results for the page from the Link project group (1).

webpage	*contrast*	*result*	*SE*	*df*	*95% CI*	*p*
Poo-Pourri	A targeted - B targeted	-2.31	1.0 0	1265.64	[-4.27, -0.34]	0.02 1

Table S4. Results for the page from the Link project group (2).

webpage	*variant*	*AOI*	*EMM*	*SE*	*df*	*95% CI*
Poo-Pourri	A	targeted	6.45	0.70	1258.06	[5.08, 7.83]
	B	targeted	8.76	0.71	1271.72	[7.36, 10.16]

1.2. Time of first fixation

1.2.1. *Icon project group*

Table S5. Results of project group Icon. Time of first fixation (1).

project group	*contrast*	*result*	*SE*	*df*	*95% CI*	*p*
Icon	A targeted - B targeted	-0.03	0.02	1278.79	[-0.07, 0.00]	0.042

Table S6. Icon project group results. Time of first fixation (2).

Project group	*variant*	*AOI*	*EMM*	*SE*	*df*	*95%CI*
Icon	A	targeted	0.23	0.01	1071.16	[0.21, 0.26]

	B	targeted	0.26	0.01	1089.01	[0.24, 0.29]

1.3. Fixation time

1.3.1. *Pages from the Icon project group*

Table S7. Results for pages in the Icon project group. Fixation time (1).

webpage	*contrast*	*result*	*SE*	*df*	*95% CI*	*p*
Films	A targeted - B targeted	0.06	0.03	1946.26	[0.11, 0.02]	0.010

Webpage	*variant*	*AOI*	*EMM*	*SE*	*df*	*95% CI*
Films	A	targeted	0.45	0.02	1943.37	[0.41, 0.48]
	B	targeted	0.38	0.02	1949.41	[0.35, 0.42]

Table S8. Results for pages in the Icon project group. Fixation time (2).

webpage	*contrast*	*result*	*SE*	*df*	*95% CI*	*p*
Green Energy	A targeted - B targeted	-0.06	0.02	1823.80	[-0.10, -0.02]	0.005

webpage	*variant*	*AOI*	*EMM*	*SE*	*df*	*95% CI*
Green Energy	A	targeted	0.34	0.01	1835.11	[0.31, 0.37]
	B	targeted	0.40	0.02	1813.34	[0.37, 0.42]

Table S9. Results for pages in the Icon project group. Fixation time (3).

webpage	*contrast*	*result*	*SE*	*df*	*95% CI*	*p*
OLX	A targeted - B targeted	-0.08	0.02	1803.28	[-0.12, -0.04]	<0.001

webpage	*variant*	*AOI*	*EMM*	*SE*	*df*	*95% CI*
OLX	A	targeted	0.36	0.01	1788.99	[0.33, 0.38]
	B	targeted	0.43	0.02	1814.67	[0.40, 0.46]

1.3.2. *Project group Contrast*

Table S10. Results of the Contrast project group. Fixation time (1).

project group	*contrast*	*result*	*SE*	*df*	*95% CI*	*p.value*
Contrast	A targeted - B targeted	-0.03	0.01	2 029.99	[-0.05, 0.00]	0.019

Table S11. Results of the Contrast project group. Fixation time (2).

Project group	*variant*	*AOI*	*EMM*	*SE*	*df*	*95%CI*
Contrast	A	targeted	0.29	0.01	1450,34	[0.27, 0.31]
Contrast	B	targeted	0.32	0.01	1475,40	[0.30, 0.34]

1.3.3. *Project group Link*

Table S12. Link project group results. Fixation time (1).

Project group	*variant*	*AOI*	*EMM*	*SE*	*df*	*95% CI*
Link	A targeted - B targeted	0.05	0.02	2042.45	[0.01, 0.09]	0.024

Table S13. Link project group results. Fixation time (2).

Project group	*variant*	*AOI*	*EMM*	*SE*	*df*	*95%CI*
Link	A	targeted	0.46	0.01	1982,27	[0.43, 0.49]
Link	B	targeted	0.41	0.01	1988,32	[0.38, 0.44]

1.3.4. *Pages from the Link project group*

Table S14. Results for pages in the Link project group. Fixation time (1).

Webpage	*contrast*	*result*	*SE*	*df*	*95% CI*	*p*
Salads	A targeted - B targeted	0.08	0.03	1972.86	[0.02, 0.13]	0.004

Table S15. Results for pages in the Link project group. Fixation time (2).

Webpage	*variant*	*AOI*	*EMM*	*SE*	*df*	*95% CI*
Salads	A	targeted	0.67	0.02	1964.10	[0.63, 0.71]
	B	targeted	0.59	0.02	1980.43	[0.55, 0.63]

1.4. Total fixation time

1.4.1. *Project group Kontrast*

Table S16. Results for the Contrast project group. Total fixation time (1).

project group	*contrast*	*result*	*SE*	*df*	*95% CI*	*p.value*
Contrast	And the search area - B search area	-1.04	0.43	2042.75	[-1.89, -0.19]	0.016

Table S17. Results for the Contrast project group. Total fixation time (2).

Project group	*variant*	*AOI*	*EMM*	*SE*	*df*	*95%CI*
Contrast	A	search area	5.55	0.33	1205.15	[4.91, 6.19]
	B	search area	6.59	0.32	1196.09	[5.96, 7.22]

1.4.2. *Pages from the Contrast project group*

Table S18. Results for pages from the Contrast project group. Total fixation time (1).

Webpage	*contrast*	*result*	*SE*	*df*	*95% CI*	*p*
InPost	A search area - B search area	-3.35	0.72	1629.60	[-4.76, -1.93]	<0.001

Table S19. Results for pages in the Contrast project group. Total fixation time (2).

Webpage	*variant*	*AOI*	*EMM*	*SE*	*df*	*95% CI*

InPost	A	search area	2.20	0.53	1621.66	[1.16, 3.23]
	B	search area	5.54	0.49	1638.62	[4.57, 6.51]

1.4.3. *Project group Link*

Table S20. Results for the Link project group. Total fixation time (1).

project group	*contrast*	*result*	*SE*	*df*	*95% CI*	*p.value*
Link	And the search area - B search area	-2.19	0.71	2034.23	[-3.58, -0.80]	0.002

Table S21. Results for the Link project group. Total fixation time (2).

Project group	*variant*	*AOI*	*EMM*	*SE*	*df*	*95%CI*
Link	A	search area	5.15	0.51	1917.06	[4.16, 6.15]
	B	search area	7.34	0.51	1926.81	[6.34, 8.35]

1.4.4. *Pages from the Link project group*

Table S22. Results for pages in the Link project group. Total fixation time (1).

Webpage	*contrast*	*result*	*SE*	*df*	*95% CI*	*p*
Poo-Pourri	A search area - B search area	-4.77	0.86	1844.53	[-6.46, -3.09]	<0.001

Table S23. Results for pages in the Link project group. Total fixation time (2).

webpage	*variant*	*AOI*	*EMM*	*SE*	*df*	*95% CI*
Poo-Pourri	A	search area	3.95	0.60	1802.89	[2.77, 5.13]
	B	search area	8.72	0.61	1881.39	[7.53, 9.92]

1.5. Visit duration

1.5.1. *Pages from the Icon project group*

Table S24. Results for pages from the Icon project group. Visit duration (1).

webpage	*contrast*	*result*	*SE*	*df*	*95% CI*	*p*
Green Energy	And the search area - B search area	1.10	0.26	1814.33	[0.58, 1.62]	<0.001

Table S25. Results for pages in the Icon project group. Visit duration (2).

webpage	*variant*	*AOI*	*EMM*	*SE*	*df*	*95% CI*
Green Energy	A	search area	4.00	0.18	1826.15	[3.64, 4.35]
	B	search area	2.89	0.19	1803.39	[2.52, 3.27]

1.5.2. *Pages from the Contrast project group*

Table S26. Results for pages in the Contrast project group. Visit duration (1).

Webpage	*contrast*	*result*	*SE*	*df*	*95% CI*	*p*
InPost	A differential - B differential	0.65	0.27	1832.39	[0.12, 1.17]	0.016

Table S27. Results for pages in the Contrast project group. Visit duration (2).

Webpage	variant	AOI	EMM	SE	df	95% CI
InPost	A	differential	2.92	0.19	1816.43	[2.54, 3.3]
	B	differential	2.27	0.19	1849.07	[1.91, 2.64]

Table S28. Results for pages in the Contrast project group. Visit duration (3).

webpage	*contrast*	*result*	*SE*	*df*	*95% CI*	*p*
Flower Delivery	A targeted - B targeted	-0.55	0.27	1819.62	[-1.07, -0.03]	0.03 8

Table S29. Results for pages in the Contrast project group. Visit duration (4).

webpage	*variant*	*AOI*	*EMM*	*SE*	*df*	*95% CI*
Flower Delivery	A	targeted	1.63	0.19	1778.49	[1.27, 2.00]
	B	targeted	2.19	0.19	1857.08	[1.82, 2.55]

1.5.3. *Pages from the Link project group*

Table S30. Results for pages in the Link project group. Visit duration (1).

Webpage	*contrast*	*result*	*SE*	*df*	*95% CI*	*p*
Poo-Pourri	A search area - B search area	-1.08	0.33	1948.93	[-1.71, -0.44]	0.00 1

Table S31. Results for pages from the Link project group. Visit duration (2).

webpage	*variant*	*AOI*	*EMM*	*SE*	*df*	*95% CI*
Poo-Pourri	A	search area	1.63	0.23	1931.36	[1.18, 2.08]
	B	search area	2.70	0.23	1963.59	[2.25, 3.16]

1.6. total visit duration

1.6.1. *Project group Contrast*

Table S32. Results for the Contrast project group. total visit duration (1).

project group	*contrast*	*result*	*SE*	*df*	*95% CI*	*p.valu e*
Contrast	And the search area - B search area	-1.15	0.50	2042.03	[-2.13, -0.18]	0.020

Table S33. Results for the Contrast project group. total visit duration (2).

Project group	*variant*	*AOI*	*EMM*	*SE*	*df*	*95%CI*
Contrast	A	search area	6.32	0.37	1251.64	[5.58, 07.05]
	B	search area	7.47	0.37	1245.70	[6.75, 8.19]

1.6.2. *Pages from the Contrast project group*

Table S34. Results for pages in the Contrast project group. total visit duration (1).

webp age	*contrast*	*result*	*SE*	*df*	*95% CI*	*p*
InPo st	A search area - B search area	-3.89	0.83	1682.58	[-5.52, -2.26]	<0.001

Table S35. Results for pages in the Contrast project group. total visit duration (2).

Webpage	variant	AOI	EMM	SE	df	95% CI
InPost	A	search area	2.55	0.61	1675.45	[1.36, 3.74]
	B	search area	6.44	0.57	1690.68	[5.33, 7.56]

1.6.3. *Link project group*

Table S36. Results for the Link project group. total visit duration (1).

project group	contrast	result	SE	df	95% CI	p.value
Link	A search area - B search area	-2.73	0.81	2036.16	[-4.32, -1.13]	0.001

Table S37. Results for the Link project group. total visit duration (2).

Project group	variant	AOI	EMM	SE	df	95%CI
Link	A	search area	06.02	0.58	1932.00	[4.88, 7.17]
	B	search area	8.75	0.59	1941.02	[7.60, 9.90]

1.6.4. *Pages from the Link project group*

Table S38. Results for pages from the Link project group. total visit duration (1).

webpage	contrast	result	SE	df	95% CI	p
Poo-Pourri	A search area - B search area	-6.00	0.99	1873.09	[-7.94, -4.06]	<0.001

Table S39. Results for pages from the Link project group. total visit duration (2).

webpage	variant	AOI	EMM	SE	df	95% CI
Poo-Pourri	A	search area	4.73	0.69	1837.23	[3.37, 6.09]
	B	search area	10.73	0.70	1904.43	[9.35, 12.11]

1.7. Time to first mouse click

1.7.1. *Pages from the Contrast project group*

Table S40. Results for pages from the Contrast project group. Time to first mouse click (1).

Webpage	contrast	result	SE	df	95% CI	p
InPost	A targeted - B targeted	-5.85	1.66	861.43	[-9.11, -2.6]	<0.001

Table S41. Results for pages from the Contrast project group. Time to first mouse click (2).

InPost	A	targeted	9.67	1.20	838.26	[7.32, 12.03]
	B	targeted	15.53	1.15	887.10	[13.28, 17.78]

1.7.2. *Pages from the Link project group*

Table S42. Results for pages from the Link project group. Time to first mouse click (1).

Webpage	contrast	result	SE	df	95% CI	p
Poo-Pourri	A targeted - B targeted	-7.81	1.97	1081.34	[-11.67, -3.95]	<0.001

Table 43. Results for pages from the Link project group. Time to first mouse click (2).

webpage	*variant*	*AOI*	*EMM*	*SE*	*df*	*95% CI*
Poo-Pourri	A	targeted	10.00	1.38	1037.02	[7.29, 12.72]
	B	targeted	17.82	1.40	1122.95	[15.08, 20.55]

1.8. Time from first fixation to mouse click

1.8.1. *Project group Contrast*

Table S44. Results for the Contrast project group. Time from first fixation to mouse click (1).

Project group	*contrast*	*result*	*SE*	*df*	*95% CI*	*p.value*
Contrast	A differential - B differential	-2.65	1.00	1303.21	[-4.62, -0.69]	0.008

Table S45. Results for the Contrast project group. Time from first fixation to mouse click (2).

Project group	*variant*	*AOI*	*EMM*	*SE*	*df*	*95%CI*
Contrast	A	differential	9.27	0.75	986.32	[7.79, 10.75]
	B	differential	11.92	0.74	979.43	[10.46, 13.38]

1.8.2. *Pages from the Contrast project group*

Table S46. Results for pages from the Contrast project group. Time from first fixation to mouse click (1).

Webpage	*contrast*	*result*	*SE*	*df*	*95% CI*	*p*
InPost	A targeted - B targeted	-4.61	1.52	1067.08	[-7.59, -1.63]	0.002
	A differential - B differential	-5.73	1.52	1067.08	[-8.71, -2.75]	<0.001

Table S47. Results for pages from the Contrast project group. Time from first fixation to mouse click (2).

Webpage	*variant*	*AOI*	*EMM*	*SE*	*df*	*95% CI*
InPost	A	targeted	8.01	1.10	1049.69	[5.86, 10.16]
	B	targeted	12.62	1.05	1085.66	[10.56, 14.68]
	A	differential	9.02	1.10	1049.69	[6.87, 11.17]
	B	differential	14.75	1.05	1085.66	[12.69, 16.81]

1.8.3. *Project group Link*

Table S48. Results for the Link project group. Time from first fixation to mouse click (1).

project group	*contrast*	*result*	*SE*	*df*	*95% CI*	*p.value*
Link	A targeted - B targeted	-2.96	1.41	1304.99	[-5.73, -0.19]	0.036
Link	A differential - B differential	-3.15	1.41	1304.99	[-5.92, -0.38]	0.026

Table S49. Results for the Link project group. Time from first fixation to mouse click (2).

Project group	*variant*	*AOI*	*EMM*	*SE*	*df*	*95%CI*

Link	A	targeted	2.63	01.02	1227.79	[0.64, 4.63]
	B	targeted	5.59	01.02	1235.56	[3.58, 7.60]
	A	differential	7.43	01.02	1227.79	[5.44, 9.42]
	B	differential	10.58	01.02	1235.56	[8.57, 12.59]

1.8.4. *Pages from the Link project group*

Table S50. Results for pages from the Link project group. Time from first fixation to mouse click (1).

webpage	*contrast*	*result*	*SE*	*df*	*95% CI*	*p*
Poo-Pourri	A targeted - B targeted	-5.51	1.82	1206.39	[-9.08, -1.93]	0.003
	A differential - B differential	-7.39	1.82	1206.39	[-10.97, -3.81]	<0.001

Table S51. Results for pages from the Link project group. Time from first fixation to mouse click (2).

webpage	*variant*	*AOI*	*EMM*	*SE*	*df*	*95% CI*
Poo-Pourri	A	targeted	3.57	1.28	1182.95	[1.06, 6.08]
	B	targeted	9.08	1.30	1226.70	[6.53, 11.62]
	A	differential	6.60	1.28	1182.95	[4.08, 9.11]
	B	differential	13.99	1.30	1226.70	[11.44, 16.53]

1.9. Total saccade time during visits

1.9.1. *Pages from the Contrast project group*

Table S52. Results for pages in the Contrast project group. Total saccade time during visits (1).

Webpage	*contrast*	*result*	*SE*	*df*	*95% CI*	*p*
InPost	A search area - B search area	-0.55	0.13	1821.93	[-0.8, -0.29]	<0.001

Table S53. Results for pages in the Contrast project group. Total saccade time during visits (2).

Webpage	*variant*	*AOI*	*EMM*	*SE*	*df*	*95% CI*
InPost	A	search area	0.35	0.09	1817.38	[0.17, 0.54]
	B	search area	0.90	0.09	1827.07	[0.73, 1.08]

1.9.2. *Pages from the Link project group*

Table S54. Results for the Link project group. Total saccade time during visits (1).

project group	*contrast*	*result*	*SE*	*df*	*95% CI*	*p.value*
Link	A search area - B search area	-0.54	0.12	2041.08	[-0.78, -0.30]	0.000

Table S55. Results for the Link project group. Total saccade time during visits (2).

Project group	*variant*	*AOI*	*EMM*	*SE*	*df*	*95%CI*
Link	A	search area	0.87	0.09	1969.41	[0.70, 1.04]
	B	search area	1.41	0.09	1976.32	[1.24, 1.58]

Table S56. Results for pages in the Link project group. Total saccade time during visits (1).

webpage	*contrast*	*result*	*SE*	*df*	*95% CI*	*p*
Poo-Pourri	A differential - B differential	-0.32	0.15	1939.46	[-0.63, -0.02]	0.036
	A search area - B search area	-1.22	0.15	1939.46	[-1.53, -0.92]	<0.001

Table S57. Results for pages in the Link project group. Total saccade time during visits (2).

webpage	*variant*	*AOI*	*EMM*	*SE*	*df*	*95% CI*
Poo-Pourri	A	differential	0.57	0.11	1919.30	[0.35, 0.78]
	B	differential	0.89	0.11	1956.42	[0.68, 1.11]
	A	search area	0.78	0.11	1919.30	[0.57, 0.99]
	B	search area	2.00	0.11	1956.42	[1.79, 2.22]

1.10. Number of fixations before

1.10.1. Pages from the Icon project group

Table S58. Results for pages from the Icon project group. Number of fixations before (1).

webpage	*contrast*	*result*	*SE*	*CI 95%*	*p*
Tchibo	A targeted - B targeted	0.34	0.10	[0.13, 0.54]	0.001

Table S59. Results for pages from the Icon project group. Number of fixations before (2).

webpage	*variant*	*AOI*	*EMM*[1]	*SE*	*95% CI*
Tchibo	A	targeted	02.09	0.07	[1.94, 2.23]
	B	targeted	1.75	0.07	[1.61, 1.90]

Table S60. Results for pages from the Icon project group. Number of fixations before (3).

webpage	*contrast*	*result*	*SE*	*CI 95%*	*p*
Green Energy	A targeted - B targeted	0.23	0.07	[0.08, 0.37]	0.002
	A differential - B differential	0.37	0.08	[0.22, 0.52]	0.000

Table S61. Results for pages from the Icon project group. Number of fixations before (4).

webpage	*variant*	*AOI*	*EMM*[2]	*SE*	*95% CI*
Green Energy	A	targeted	3.65	0.05	[3.56, 3.75]

[1] Logarithm of values

[2] Logarithm of values

	B	targeted	3.43	0.05	[3.32, 3.53]
	A	differential	3.37	0.05	[3.28, 3.47]
	B	differential	3.01	0.06	[2.89, 3.12]

Table S62. Results for pages in the Icon project group. Number of fixations before (5).

webpage	*contrast*	*result*	*SE*	*CI 95%*	*p*
OLX	A targeted - B targeted	-0.19	0.08	[-0.34, -0.03]	0.018
	A differential - B differential	-0.58	0.22	[-1.01, -0.15]	0.008

Table S63. Results for pages in the Icon project group. Number of fixations before (6).

webpage	*variant*	*AOI*	*EMM*[3]	*SE*	*95% CI*
OLX	A	differential	2.92	0.05	[2.82, 03.03]
	B	differential	3.11	0.06	[3.00, 3.22]
	A	targeted	-0.25	0.17	[-0.58, 0.07]
	B	targeted	0.32	0.14	[0.05, 0.60]

Table S64. Results for pages from the Icon project group. Number of fixations before (7).

webpage	*contrast*	*result*	*SE*	*CI 95%*	*p*
Shopping area	A targeted - B targeted	0.42	0.11	[0.20, 0.64]	0.000

Table S65. Results for pages from the Icon project group. Number of fixations before (8).

webpage	*variant*	*AOI*	*EMM*[4]	*SE*	*95% CI*
Shopping area	A	targeted	2.22	0.07	[2.08, 2.35]
	B	targeted	1.79	0.09	[1.62, 1.97]

1.10.2. *Pages from the Contrast project group*

Table S66. Results for pages in the Contrast project group. Number of fixations before (1).

webpage	*contrast*	*result*	*SE*	*CI 95%*	*p*
Fitness Blender	A targeted - B targeted	-0.40	0.09	[-0.58, -0.22]	0.000

Table S67. Results for pages in the Contrast project group. Number of fixations before (2).

webpage	*variant*	*AOI*	*EMM*[5]	*SE*	*95% CI*
Fitness Blender	A	targeted	2.10	0.07	[1.96, 2.24]
	B	targeted	2.50	0.06	[2.38, 2.62]

Table S68. Results for pages in the Contrast project group. Number of fixations before (3).

[3] Logarithm of values
[4] Logarithm of values
[5] Logarithm of values

webpage	contrast	result	SE	CI 95%	p
InPost	A targeted - B targeted	-0.63	0.11	[-0.85, -0.41]	0.000

Table S69. Results for pages in the Contrast project group. Number of fixations before (4).

webpage	variant	AOI	EMM[6]	SE	95% CI
InPost	A	targeted	1.48	0.09	[1.31, 1.65]
	B	targeted	2.11	0.07	[1.98, 2.25]

Table S70. Results for pages in the Contrast project group. Number of fixations before (5).

webpage	contrast	result	SE	CI 95%	p
Flower Delivery	A targeted - B targeted	-0.34	0.09	[-0.52, -0.17]	0.000

Table S71. Results for pages in the Contrast project group. Number of fixations before (6).

webpage	variant	AOI	EMM[7]	SE	95% CI
Flower Delivery	A	targeted	2.31	0.07	[2.18, 2.44]
	B	targeted	2.65	0.06	[2.54, 2.77]

Table S72. Scores for pages in the Contrast project group. Number of fixations before (7).

webpage	contrast	result	SE	CI 95%	p
Sunny Spain	A targeted - B targeted	0.19	0.08	[0.03, 0.35]	0.019
	A differential - B differential	0.41	0.11	[0.20, 0.62]	0.000

Table S73. Results for pages in the Contrast project group. Number of fixations before (8).

webpage	variant	AOI	EMM[8]	SE	95% CI
Sunny Spain	A	targeted	4.02	0.06	[3.91, 4.13]
	B	targeted	3.83	0.06	[3.71, 3.95]
	A	differential	3.04	0.07	[2.90, 3.18]
	B	differential	2.63	0.08	[2.47, 2.79]

1.11. Number of fixations

1.11.1. Pages from the Icon project group

Table S74. Results for pages from the Icon project group. Number of fixations (1).

webpage	contrast	result	SE	CI 95%	p
Films	A differential - B differential	-0.18	0.08	[-0.33, -0.03]	0.018

[6] Logarithm of values
[7] Logarithm of values
[8] Logarithm of values

Table S75. Results for pages in the Icon project group. Number of fixations (2).

webpage	*variant*	*AOI*	*EMM*[9]	*SE*	*95% CI*
Films	A	differential	3.25	0.06	[3.14, 3.36]
	B	differential	3.43	0.05	[3.33, 3.53]

Table S76. Results for pages in the Icon project group. Number of fixations (3).

webpage	*contrast*	*result*	*SE*	*CI 95%*	*p*
Shopping area	A search area - B search area	0.20	0.09	[0.03, 0.37]	0.022

Table S77. Results for pages in the Icon project group. Number of fixations (4).

webpage	*variant*	*AOI*	*EMM*[10]	*SE*	*95% CI*
Shopping area	A	targeted	2.03	0.07	[1.88, 2.17]
	B	targeted	2.13	0.08	[1.98, 2.28]
	A	differential	2.83	0.06	[2.72, 2.94]
	B	differential	2.88	0.06	[2.76, 3.03]
	A	search area	2.85	0.06	[2.74, 2.96]
	B	search area	2.65	0.06	[2.52, 2.78]

1.11.2. *Pages from the Contrast project group*

Table S78. Results for pages in the Contrast project group. Number of fixations (1).

webpage	*contrast*	*result*	*SE*	*CI 95%*	*p*
Fitness Blender	A targeted - B targeted	0.21	0.09	[0.04, 0.39]	0.018

Table S79. Results for pages in the Contrast project group. Number of fixations (2).

webpage	*variant*	*AOI*	*EMM*[11]	*SE*	*95% CI*
Fitness Blender	A	targeted	2.33	0.06	[2.20, 2.46]
	B	targeted	2.12	0.06	[1.99, 2.25]
	A	differential	2.91	0.05	[2.80, 3.02]
	B	differential	2.80	0.05	[2.72, 2.92]
	A	search area	2.79	0.06	[2.72, 2.85]
	B	search area	2.81	0.05	[2.71, 2.91]

Table S80. Results for pages in the Contrast project group. Number of fixations (3).

webpage	*contrast*	*result*	*SE*	*CI 95%*	*p*
InPost	A search area - B search area	-0.84	0.08	[-1.00, -0.68]	<0.001

Table S81. Results for pages in the Contrast project group. Number of fixations (4).

webpage	*variant*	*AOI*	*EMM*[12]	*SE*	*95% CI*
InPost	A	targeted	2.75	0.06	[2.64, 2.87]
	B	targeted	2.78	0.05	[2.67, 2.89]

[9] Logarithm of values

[10] Logarithm of values

[11] Logarithm of values

[12] Logarithm of values

	A	differential	3.29	0.05	[3.19, 3.39]
	B	differential	3.41	0.05	[3.31, 3.50]
	A	search area	2.32	0.07	[2.19, 2.45]
	B	search area	3.61	0.05	[3.06, 3.26]

Table S82. Results for pages in the Contrast project group. Number of fixations (5).

webpage	*contrast*	*result*	*SE*	*CI 95%*	*p*
Sunny Spain	A search area - B search area	0.14	0.08	[-0.01, 0.29]	0.00 8

Table S83. results for pages in the Contrast project group. Number of fixations (6).

webpage	*variant*	*AOI*	*EMM*[13]	*SE*	*95% CI*
Sunny Spain	A	targeted	0.89	0.16	[0.58, 1.20]
	B	targeted	0.96	0.16	[0.65, 1.27]
	A	differential	2.78	0.07	[2.64, 2.93]
	B	differential	2.94	0.07	[2.80, 3.08]
	A	search area	3.84	0.05	[3.73, 3,95]
	B	search area	3.70	0.06	[3.59, 3.82]

1.11.3. *Link project group*

Table S84. Results for the Link project group. Fixation time (1).

project group	*contrast*	*result*	*SE*	*CI 95%*	*p*
Link	A search area - B search area	-0.26	0.12	[-0.50, -0.01]	0.039

Table S85. Results for the Link project group. Fixation time (2).

project group	*variant*	*AOI*	*EMM*[14]	*SE*	*95% CI*
Link	A	search area	3.15	0.09	[2.97, 3.33]
	B	search area	3.41	0.09	[3.23, 3.58]

1.11.4. *Pages from the Link project group*

Table S86. Results for pages in the Link project group. Fixation time (1).

webpage	*contrast*	*result*	*SE*	*CI 95%*	*p*
Poo-Pourri	A targeted - B targeted	-0.27	0.11	[-0.49, -0.06]	0.014
	A differential - B differential	-0.19	0.09	[-0.35, -0.02]	0.029
	A search area - B search area	-0.74	0.08	[-0.89, -0.58]	<0.001

Table S87. Results for pages in the Link project group. Fixation time (2).

webpage	*variant*	*AOI*	*EMM*[15]	*SE*	*95% CI*

[13] Logarithm of values

[14] Logarithm of values

[15] Logarithm of values

Poo-Pourri	A	targeted	1.92	0.08	[1.76, 2.09]
	B	targeted	2.20	0.07	[2.06, 2.34]
	A	differential	2.81	0.06	[2.69, 2.94]
	B	differential	3.00	0.06	[2.89, 3.11]
	A	search area	2.91	0.06	[2.80, 3.04]
	B	search area	3.65	0.05	[3.55, 3.75]

Table S88. Results for pages in the Link project group. Fixation time (3).

webpage	*contrast*	*result*	*SE*	*CI 95%*	*p*
Salads	A search area - B search area	0.17	0.08	[0.01, 0.33]	0.04 0

Table S89. Results for pages in the Link project group. Fixation time (4).

webpage	*variant*	*AOI*	*EMM*[16]	*SE*	*95% CI*
Salads	A	search area	3.31	0.06	[3.20, 3.42]
	B	search area	3.14	0.06	[3.03, 3.26]

1.12. Number of visits

1.12.1. *Project group Contrast*

Table S90. Results for the Contrast project group. Number of visits (1).

project group	*contrast*	*result*	*SE*	*CI 95%*	*p*
Contrast	A differential - B differential	-0.24	0.08	[-0.39, -0.09]	0.002

Table S91. Results for the Contrast project group. Number of visits (2).

Project group	*variant*	*AOI*	*EMM*	*SE*	*95%CI*
Contrast	A	differential	1.17	0.06	[1.05, 1.29]
	B	differential	1.41	0.05	[1.31, 1.52]

1.12.2. *Pages from the Contrast project group*

Table S92. results for pages from the Contrast project group. Number of visits (1).

webpage	*contrast*	*result*	*SE*	*CI 95%*	*p*
InPost	A differential - B differential	-0.33	0.13	[-0.58, -0.08]	0.009
	A search area - B search area	-0.58	0.14	[-0.83, -0.30]	<0.001

Table S93. Results for pages from the Contrast project group. Number of visits (2).

webpage	*variant*	*AOI*	*EMM*[17]	*SE*	*95% CI*
InPost	A	differential	1.11	0.10	[0.92, 1.30]
	B	differential	1.43	0.08	[1.28, 1.60]
	A	search area	0.89	0.11	[0.67, 1.10]
	B	search area	1.46	0.08	[1.30, 1.61]

[16] Logarithm of values

[17] Logarithm of values

Table S94. results for pages from the Contrast project group. Number of visits (3).

webpage	*contrast*	*result*	*SE*	*CI 95%*	*p*
Sunny Spain	A differential - B differential	-0.37	0.16	[-0.68, -0.07]	0.01 7

Table S95. results for pages from the Contrast project group. Number of visits (4).

webpage	*variant*	*AOI*	*EMM*[18]	*SE*	*95% CI*
Sunny Spain	A	differential	1.56	0.12	[1.33, 1.80]
	B	differential	1.93	0.11	[1.73, 2.14]

1.12.3. *Pages from the Link project group*

Table S96. Results for pages from the Link project group. Number of visits (1).

webpage	*contrast*	*result*	*SE*	*CI 95%*	*p*
Poo-Pourri	A search area - B search area	-0.33	0.16	[-0.64, -0.03]	0.031

Table S97. Results for pages from the Link project group. Number of visits (2).

webpage	*variant*	*AOI*	*EMM*[19]	*SE*	*95% CI*
Poo-Pourri	A	search area	1.06	0.12	[0.82, 1.29]
	B	search area	1.40	0.10	[1.19, 1.60]

[18] Logarithm of values

[19] Logarithm of values
